# The RNA-binding protein GRSF1 promotes hepatocarcinogenesis via competitively binding to YY1 mRNA with miR-30e-5p

**DOI:** 10.1186/s13046-021-02217-w

**Published:** 2022-01-08

**Authors:** Lili Han, Chen Huang, Xiaofei Wang, Dongdong Tong

**Affiliations:** 1 Department of Oncology, The Second Affiliated Hospital, College of Medicine, Xi’an Jiaotong University, Xi’an, 710004 Shaanxi China; 2 Key Laboratory of Environment and Genes Related to Diseases, Ministry of Education of China, Xi’an Jiaotong University, No.277 Yanta West Road, Xi’an, 710061 Shaanxi Province China

**Keywords:** RBP, GRSF1, YY1, HCC, miR-30e-5p

## Abstract

**Background:**

Dysregulation of RNA binding protein (RBP) expression has been confirmed to be causally linked with tumorigenesis. The detailed biological effect and underlying mechanisms of the RBP GRSF1 in hepatocellular carcinoma (HCC) remain unclear.

**Methods:**

HCC cells with stable knockdown of GRSF1 were established using two sh-RNA-encoding lentiviruses. The functions of GRSF1 in HCC were explored using MTT, colony formation, flow cytometry, and Transwell assays and a xenograft model. Transcriptomic sequencing in GRSF1-deficient MHCC-97H cells was carried out to identify the downstream effector of GRSF1. The regulatory mechanisms among GRSF1, YY1 and miR-30e-5p were investigated via RNA immunoprecipitation, luciferase, RNA pull-down and ChIP assays. Several in vivo assays were used to assess the selectivity of the small-molecule compound VE-821 in HCC and to confirm the absence of general toxicity in animal models.

**Results:**

GRSF1 was frequently increased in HCC tissue and cells and was associated with worse clinical outcomes. GRSF1 functions as a novel oncogenic RBP by enhancing YY1 mRNA stability, and the GUUU motifs within the YY1 3`UTR 2663-2847 were the specific binding motifs for GRSF1. YY1 feedback promoted GRSF1 expression by binding to the GRSF1 promoter. In addition, YY1 was a critical target of miR-30e-5p, which was confirmed in this study to inhibit HCC hepatocarcinogenesis. GRSF1 and miR-30e-5p competitively regulated YY1 by binding to its 3`UTR 2663-2847 region. Finally, we identified that VE-821 blocked HCC progression by inhibiting the GRSF1/YY1 pathway.

**Conclusion:**

This study revealed the interaction network among GRSF1, YY1 and miR-30e-5p, providing new insight into HCC pathogenesis, and indicated that VE821 may serve as a novel agent with potential for HCC treatment through inhibition of the GRSF1/YY1 axis.

**Supplementary Information:**

The online version contains supplementary material available at 10.1186/s13046-021-02217-w.

## Background

Hepatocellular carcinoma (HCC) is one of the most commonly diagnosed cancers worldwide and has a poor prognosis [1, 2]. Despite intensive efforts to improve therapeutic strategies, HCC patients still face unsatisfactory prognoses [3]. Therefore, exploring HCC pathogenesis and identifying new effective therapeutic targets are of great interest.

RNA binding proteins (RBPs) are of immense importance in diverse biological regulatory processes, including RNA splicing, degradation, stabilization, modification and translation [4–7]. Mounting evidence indicates that dysregulation of RBPs contributes to transcriptomic imbalance and thus drives tumorigenicity [8–13]. G-rich sequence binding factor 1 (GRSF1), a recently identified RBP, has a significant impact on almost all steps of posttranscriptional regulation by binding to mRNAs through its three RRM (RNA-binding) domains. GRSF1 is essential in preventing premature senescence induced by oxidative stress [14]. Recent observations have implicated GRSF1 in cancer progression. GRSF1 drives the metastasis of cervical cancer cells via the PIK3R3/AKT/NF-κB and TIMP3/MMP9 pathways [15]. In addition, GRSF1 was revealed to promote cervical cancer by enhancing TMED5 and LMNB1 expression [16]. However, far less is known about the potential function of GRSF1 in HCC. The current study is the first to discover that GRSF1 is frequently increased in HCC and promotes hepatocarcinogenesis as an RBP.

Yin-Yang 1 (YY1), which can act as a transcriptional activator of oncogenes or a repressor of cancer suppressors, is a critical promoter of hepatocarcinogenesis [17–19]. Extensive evidence confirms that YY1 expression is significantly increased in HCC tissue and is closely correlated with the expression of other cancer-related genes. However, the regulatory mechanism upstream of YY1 in HCC remains unknown. This study identified YY1 as an essential downstream effector of GRSF1 and rescued the tumor-inhibiting effect of GRSF1 knockdown, suggesting that GRSF1 promotes hepatocarcinogenesis by promoting YY1 expression.

MicroRNAs (miRNAs or miRs) are a group of noncoding RNAs that are 18-24 nucleotides in length. MicroRNAs are involved in regulating various biological processes in HCC [20–22]. miR-30e-5p, a novel cancer-related miRNA, is involved in the progression of various human cancers. miR-30e-5p was reported to inhibit nasopharyngeal carcinoma migration by repressing MAT1 [23], impair nonsmall cell lung carcinoma growth by suppressing its downstream effectors [24], and prevent bladder cancer tumorigenesis by inhibiting MTDH [25]. However, the role of miR-30e-5p in HCC is less well reported. In this study, we uncovered that miR-30e-5p acts as a tumor suppressor in HCC by regulating YY1 as an upstream regulator competing with GRSF1.

In conclusion, we discovered that the RBP GRSF1 promoted HCC tumorigenesis in vitro and in vivo by enhancing YY1 stability. The GUUU motifs within the YY1 3`UTR 2663-2847 were the specific binding motifs for GRSF1. YY1 activated the GRSF1 promoter and enhanced its expression, forming a feedback loop. GRSF1 competitively regulated YY1 by binding to its 3`UTR mRNA with miR-30e-5p. Finally, we identified that VE-821, a small-molecule compound, blocked HCC progression by inhibiting the GRSF1/YY1 pathway, providing a novel potential treatment option for HCC.

## Materials and methods

### Patient tissue specimens

The ethics committee of Xi’an Jiaotong University approved this study (Approval number: 130043). A total of 120 HCC samples and paired noncancerous tissues were obtained from HCC patients during the resection of HCC lesions. None of the patients received radiotherapy, immunotherapy or chemotherapy before surgery.

### Quantitative real-time PCR

Total RNA was extracted from HCC cells or tissues using TRIzol (Invitrogen, Carlsbad, CA, USA). RNA samples were reverse transcribed into cDNA using a TaKaRa Reverse Transcription System (Dalian, China). qRT-PCR was performed using a SYBR Premix kit (Takara Bio, Inc.). The RNA level was expressed as a relative result via the 2^–ΔΔCT^ method against the endogenous standard control GAPDH or U6. The primer sequences are shown in Supplementary Table 1 (Table S1).

### Western blotting

Protein was obtained by applying RIPA buffer (Beyotime, China) and quantified using a BCA protein assay kit (Thermo Fisher, USA). Protein samples were separated via SDS–PAGE and transferred to PVDF membranes (Millipore, Billerica, MA), followed by blocking in 5% nonfat milk. After incubation with primary antibodies against GRSF1 (ab241400, Abcam), YY1 (#63227, CST) or GAPDH (ab6922; Abcam) overnight at 4 °C, the membranes were probed with secondary antibodies for 1~2 h. The data were collected using an ECL blotting analysis system (Amersham Pharmacia Biotech, Piscataway, NJ).

### Cell lines and cell culture

Human HCC cells were purchased from FuHeng Cell Shanghai Center (China), and THLE-2 immortalized human liver cells were purchased from ATCC (Manassas, VA, USA). All cells were maintained in DMEM (Sigma–Aldrich; Merck KGaA) with 10% FBS (Biological Industries, CT, USA) and cultured at 37 °C with 5% CO_2_.

### Cell transfection

Lentiviruses encoding shRNAs targeting GRSF1 or YY1, lentivirus vectors to induce GRSF1 or YY overexpression, and the corresponding negative control vectors were purchased from GenePharma (Shanghai, China). HCC cells were cultured in 6-well plates until reaching 50%-60% confluence and then were transfected with these lentivirus vectors. The medium was replaced with fresh normal medium after 6 h. After 48 h, the transfection efficiency was assessed.

### Cell migration assay

Transwell chambers with 8-μm pore size polycarbonate membranes were used to detect the migration ability of cells. Briefly, 200 μL FBS-free DMEM containing 1×10^4^ HCC cells was added to the upper chamber, and 600 μL DMEM supplemented with 10 % FBS was added to the bottom of the chamber. After 24 h, cells left on the upper surface of the membrane were removed, while those that penetrated the membrane were fixed in 4% paraformaldehyde for 30 min and stained with crystal violet. Photographs of 3 random fields were captured under an optical microscope (Wetzlar, Germany, ×200 magnification). ImageJ(v1.8.0) was used for quantification analysis.

### Apoptosis assay

HCC cells were plated in 12-well plates (1-3×10^5^ cells/well). After 24 h, they were treated with sh-GRSF1, ov-YY1 vector or VE821. After 24-48 h, the cells were digested, collected, and resuspended in 1× binding buffer. Then, the cells were treated with Annexin V-FITC and kept out of the light at room temperature for 15 minutes. The cells were mixed with PI staining solution and incubated on ice without light. After 5 minutes, cell apoptosis was analyzed via flow cytometry (EPICS, Xl-4; Beckman Coulter, Inc., USA).

### RNA immunoprecipitation (RIP assay)

Protein A Sepharose beads were washed and resuspended in NT2 buffer (50 mM Tris HCl, 150 mM NaCl, 1 mM MgCl2 and 0.05% Nonidet P-40). The antibody-coated beads were washed and resuspended in ice-cold NT2. A total of 1 x 10^7^cells (MHCC-97H, Hep3B, MHCC-97H-ov-GRSF1 or Hep3B-ov-GRSF1) were mixed with RIP lysis buffer (50 mM Tris, 5 mM EDTA, 0.5% NP-40, 100 mM NaCl,100 mM KCl, 5 mM MgCl2) supplemented with an RNase inhibitor and a protease inhibitor cocktail and incubated at 4 °C for 20 min. The precleared cell lysate was incubated with the antibody coated beads at 4 °C overnight with rotation. Then, the mixture was incubated in NT2 buffer treated with SDS, RNAse OTU and proteinase K for 2 h at room temperature. RNA was extracted according to the TRIzol manufacturer’s instructions and purified using phenol, chloroform and isoamyl alcohol. For reverse transcription, 5 μl of the RNA solution was used. For cDNA synthesis, the thermal cycler parameters were 25 °C for 10 min, 50 °C for 30 min and 5 min at 85 °C. PCR was performed with Power SYBR Green master mix following the manufacturer's protocol using a primer mix for YY1, LMNB1 or the housekeeping protein (UBC or GAPDH) mRNAs.

### Biotinylated RNA pull-down assay

Different PCR fragments of YY1 mRNA, as well as the M1, M2 and M3 mutants, were used as templates for transcription. Biotin-CTP (Promega) and T7 polymerase (Promega) were used to prepare biotinylated transcripts. The mixture of biotinylated transcripts, cell lysates and TENT buffer was incubated at room temperature for 2 h. The complexes were collected with paramagnetic streptavidin-conjugated Dynabeads (Invitrogen). Western blotting was performed to measure the pull-down materials using antibodies against GRSF1 and GAPDH.

### Luciferase reporter assay

The sequences of different YY1 mRNA fragments were amplified and cloned into a luciferase reporter vector. The corresponding empty control plasmid (pGL3-control) and the transcriptional activity control plasmid with the Renilla luciferase gene (phrL-TK) were prepared for purification and reserve. HEK293 cells were treated in 96-well plates and cultured for 12 hours. Then, the HEK293 cells were cotransfected with the reporter gene or pGL3-control, sh-GRSF1 or sh-Ctrl, pre-miR-30e-5p or pre-NC, and phRL-TK. After 48 h, the cells were washed with PBS, PLB lysate was added, and the cells were incubated at room temperature for 15 min. LAR II (a mixture of Luciferase Assay Reagent II and Luciferase Assay Substrate) was added, and the firefly luciferase value was assessed immediately using a microplate reader. Then, the reaction of LAR II was terminated by addition of a mixture of Stop&Glo® Substrate and Stop&Glo® Buffer, and Renilla luciferase activity was detected using the microplate reader. The relative luciferase/Renilla luciferase fluorescence intensity in each tube was calculated and compared with that in the control group.

### Transcriptome sequencing assays

RNA was isolated and purified from MHCC-97H GRSF1-deficient cells and control cells as described previously. RNA quantity and purity were quantified using a NanoDrop ND-1000 spectrophotometer (NanoDrop, Wilmington, USA). After deletion of low-quality bases (quality < 20) and sequencing adapters, 148,476,367,800 clean reads were analyzed. First, hisat2-2.1.0 mapped clean reads to the Human Reference genome (HG19). Then, stringtie-1.3.3 was used to assemble, merge and calculate the expression level of the transcripts. The preDE. py script was used to calculate the expression quantity based on raw count. Then, the R package DEseq2 was used to homogenize the raw count and analyze the differential expression. The overall process was hisat2 + stringtie + DEseq2. The threshold was *p* value < 0.05 and │log2 fold change│≥2. The raw data has been deposited (PRJCA007380) in Genome Sequence Archive (https://ngdc.cncb.ac.cn/gsa-human/) in National Genomics Data Center under accession codes HRA001615.

### Reporter constructs

First, YY1 3`UTR mutations in the GRSF1-binding site (YFP-YY1-GRSF1 MT) or the miR-30e-5p-binding site (YFP-YY1-miR-30e-5p MT) were constructed using an Agilent Technologies site-directed mutagenesis kit (Palo Alto, CA, USA). Subsequently, the YY1 3`UTR (YFP-YY1 WT) and the mutations were amplified and then introduced into the pd2EYFP-N1 reporter (YFP) vector (Clontech, Palo Alto, CA, USA). The sequences used for amplification are shown in Supplementary Table 2 (Table S2).

### Chromatin immunoprecipitation (ChIP)

YY1-HA tag fusion expression vectors constructed and provided by Thermo Fisher Scientific Inc. were used to detect the binding of exogenous YY1 to the GRSF1 promoter via ChIP-qPCR. ChIP assays were carried out using an Enzymatic Chromatin IP Kit (Thermo Fisher Scientific, Rockford, IL). HCC cells were treated with formaldehyde to cross link the protein to the DNA. Glycine was added to the suspension to stop fixation. Cells were harvested and suspended in ChIP lysis buffer. The cell lysate was sonicated (Branson sonifier 250, 30–40% output, 7 s eight times). The average DNA fragment size was approximately 350-450 bp. The collected lysate was treated with rabbit normal IgG (ab172730) or specific antibodies, including anti-HA tag (ab9110) and anti-RNA polymerase II Rpb1 (1509Y) antibodies. After digestion with micrococcal nuclease, immunoprecipitation samples were mixed with protein A/G magnetic beads (Millipore) and rotated overnight at 4 °C. The beads were collected, washed and then treated with extraction buffer for 12 h at room temperature to reverse the cross-linking. After digestion with Proteinase K, DNA was extracted using chloroform. The purified DNA was analyzed via qRT–PCR. For the ChIP assays to assess the binding of endogenous GRSF1 to endogenous YY1, normal rabbit IgG (#2729 ) and anti-YY1 ( #46395) antibodies were used.

### Small-molecule compound

The small-molecule compound VE821 (C18H16N4O3S, molecular weight 368.4, CAS No. 1232410-49-9) was obtained from the Department of Chemistry, Xi'an Jiaotong University. High-performance liquid chromatography determined that the purity of VE821 was more than 97%.

### Protein degradation analysis

After treatment with or without VE821 for 48 h, MHCC-97H cells in different dishes were treated with cycloheximide (CHX, 50 g/ml). At the indicated time points, protein samples were collected and then analyzed via western blotting.

### Animal model

Five-week-old male immunodeficient mice were purchased from the Animal Center of Xi'an Jiaotong University and kept in a specific pathogen-free environment. MHCC-97H cells transfected with sh-Ctrl, sh-GRSF1, pre-NC, pre-miR-30e-5p, pre-miR-30e-5p+ov-YY1, premiR-30e-5p+sh-GRSF1 or premiR-30e-5p+ov-GRSF1 vectors or precursor cells (1×10^7^) in 150 μl of serum-free medium were injected into the right flank of the mice. After tumor xenografts could be observed, the tumor sizes were measured every other day using the formula length×width^2^×0.5. For the two drug treatment groups (10 mice/group), mice received intraperitoneal injections of vehicle or VE821 (15 mg/kg) twice a week for 20 days. After 30 days, the eyes of immunodeficient mice were removed for blood collection. Blood examinations were performed using automatic hematology analyzers (BC-2800Vet, Chemray800). Then, the mice were sacrificed, and the tumors in the xenograft models were surgically removed for further analysis.

### Immunohistochemistry (IHC)

Immunohistochemical staining was performed using a diaminobenzidine detection kit (Maixin-Bio, Fuzhou, China) following the manufacturer’s instructions. Tissue sections were deparaffinized, rehydrated and incubated in goat serum. Sections were incubated with a primary antibody at room temperature for 1.5 h. After incubation with secondary antibody for 15 min, the sections were counterstained with hematoxylin, mounted with Permount and examined via light microscopy. Primary antibodies against GRSF1, YY1, and Ki67 and the corresponding secondary antibodies were all obtained from Beijing Biosynthesis Biotechnology Co., Ltd.

### Statistical analysis

All statistical analyses in this study were conducted with SPSS. Each experiment was repeated in triplicate, and the values are expressed as mean± SEM. Chi-square tests were used to identify the association between HCC clinicopathological features and GRSF1. Kaplan–Meier survival analysis with the log-rank test was used to assess the correlation between the outcome of HCC patients and GRSF1 expression. To compare the differences, Student’s t-test was used between two groups, and one-way ANOVA was used among more than two groups. *p<0.05 with a two-sided test was considered statistically significant.

## Results

### GRSF1 is increased in HCC and associated with poor prognosis

**Fig. 1:**
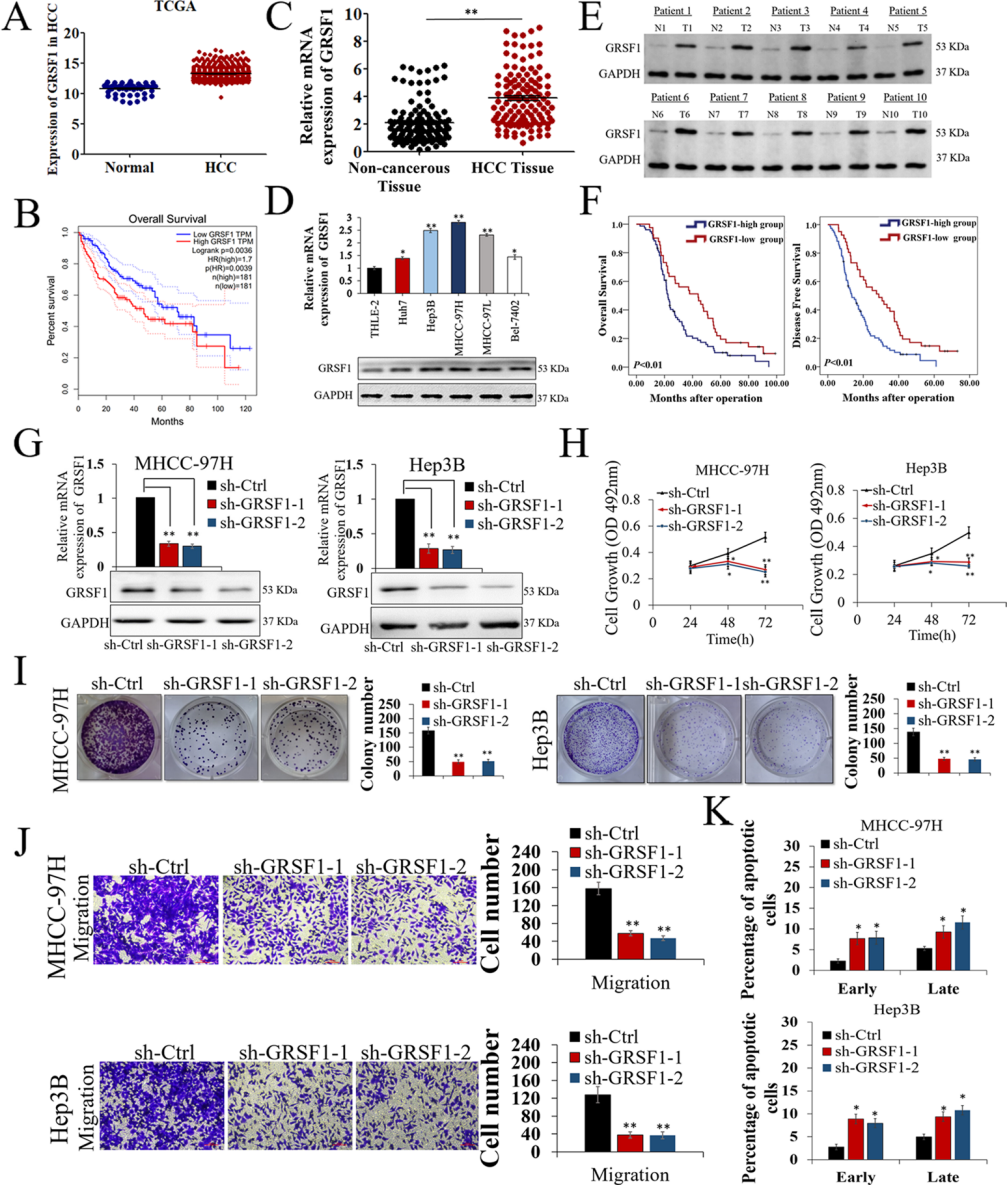
GRSF1 is frequently increased in HCC and promotes HCC in vitro. **A** Analyses of GRSF1 expression in HCC and nontumor tissues in TCGA datasets. **B** Correlation between GRSF1 expression levels and prognosis for HCC patients in TCGA datasets. **C** Analyses of GRSF1 mRNA expression levels in 120 HCC and paired noncancerous tissues. **D** GRSF1 expression levels in HCC cell lines and THLE-2 cells. **E** GRSF1 protein expression in 10 HCC samples and paired noncancerous tissues. **F** Kaplan–Meier analysis showing the association between GRSF1 expression and prognosis in 120 HCC patients. **G** GRSF1 expression levels in MHCC-97H and Hep3B cells were decreased following transfection with sh-GRSF1 lentivirus. **H-J** MTT (**H**), colony formation (**I**), and Transwell **(J**) assays showed that GRSF1 knockdown suppressed HCC cell proliferation, colony-forming ability and migration. The invaded cells in Transwell assays were quantified by counting the cells in 10 random fields (magnification, 200×). **K** FCM assays showed that GRSF1 knockdown enhanced MHCC-97H and Hep3B cell apoptosis. Values are the mean± SEM (*n *= 3); *p < 0.05, **p < 0.01

Analysis of public datasets (Cancer Genome Atlas, TCGA) suggested that GRSF1 expression was elevated in HCC tissues compared with normal tissues (*p*<0.01, Fig. 1A). Survival analysis using data from TCGA revealed that higher GRSF1 expression indicated a shorter overall survival (OS) for HCC patients (*p*<0.01, Fig. 1B). We further measured GRSF1 expression in 120 pairs of HCC tissues and adjacent nontumor clinical samples. qRT–PCR assays demonstrated that GRSF1 was obviously higher in HCC tissues (*p*<0.01, Fig. 1C). GRSF1 expression levels were also markedly increased in HCC cell lines compared with THLE-2 immortalized human liver cells (Fig. 1D, *p*<0.05). Western blot assays using samples from 10 HCC patients showed that GRSF1 expression in HCC tissues was increased compared with that in matched noncancerous tissues (*p*<0.05, Fig. 1E). Kaplan–Meier survival analysis revealed that patients with higher GRSF1 expression tended to have worse outcomes, while those with lower GRSF1 expression had better outcomes (*p*<0.01, Fig. 1F). Furthermore, chi-square tests suggested that GRSF1 upregulation was positively correlated with larger tumor size (*p*<0.01), worse differentiation (*p*<0.05), microscopic vascular invasion (*p*<0.01) and advanced TNM stage in HCC (*p*<0.01, Table S3). In short, increased GRSF1 expression in HCC was associated with an advanced stage of carcinogenesis and a worse patient prognosis.

### GRSF1 promotes HCC cell proliferation and migration

Stable silencing of GRSF1 was established in MHCC-97H and Hep3B cells using two different effective sh-RNA-encoding lentiviruses (sh-GRSF1-1 and sh-GRSF1-2). The knockdown efficiency was confirmed by qRT–PCR and western blotting assays (*p*<0.05, Fig. 1G). Functionally, MTT, colony-forming and Transwell assays revealed that GRSF1 knockdown reduced MHCC-97H and Hep3B cell proliferation (*p*<0.05, Fig. 1H), colony formation (*p*<0.01, Fig. 1I), and migration ability (*p*<0.01, Fig. 1J). To measure whether decreased GRSF1 could inhibit HCC cell proliferation by enhancing cell apoptosis, flow cytometry (FCM) assays were performed. The results showed that GRSF1 knockdown increased HCC cell apoptosis, suggesting that GRSF1 can promote cell proliferation by inhibiting cell apoptosis (*p*<0.05, Fig. 1K). These data revealed that GRSF1 promotes HCC malignant biological behavior in vitro.

### GRSF1 promotes YY1 expression by directly binding to the YY1 3`UTR

**Fig. 2:**
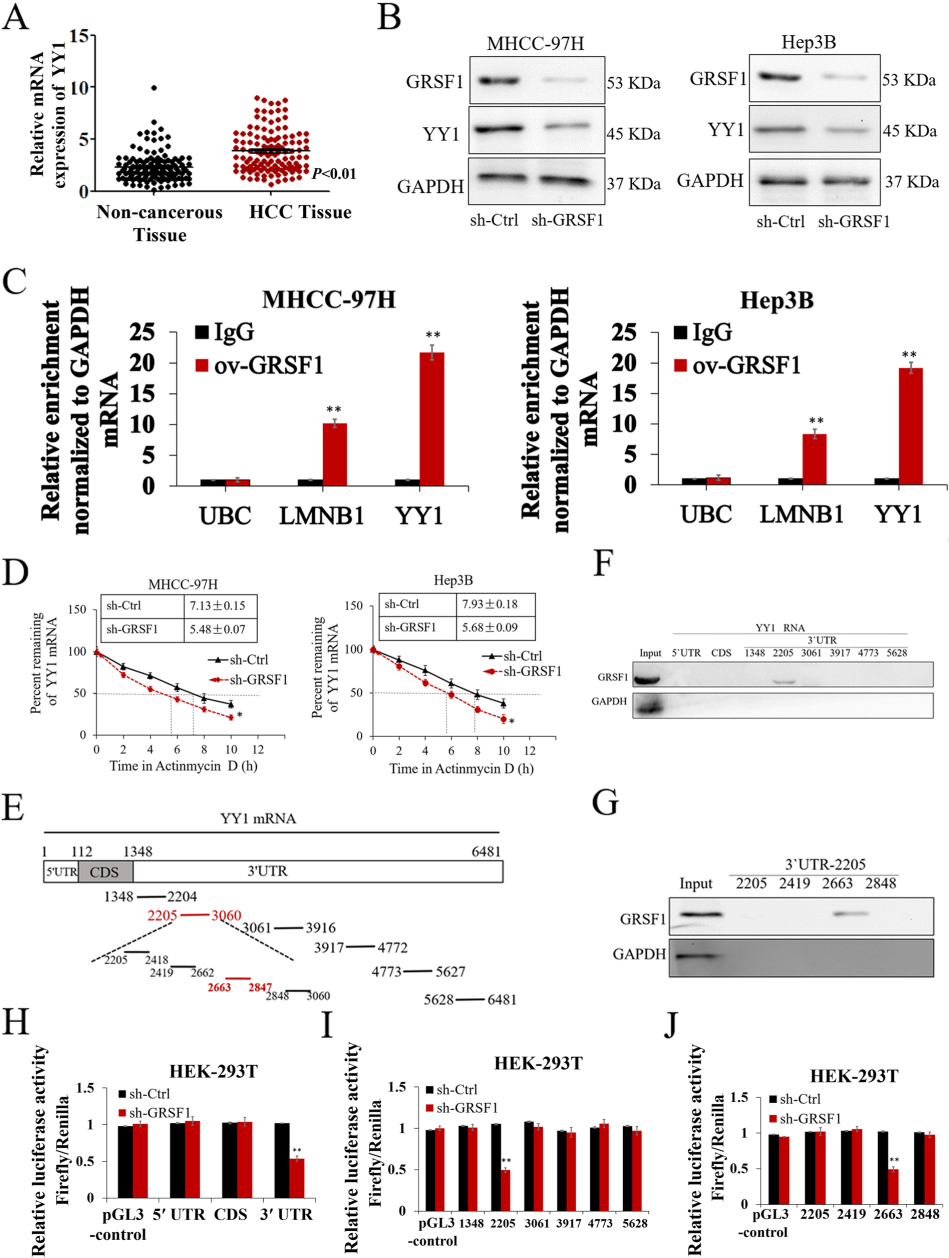
GRSF1 enhanced YY1 expression by interacting with YY1 mRNA. **A** YY1 mRNA expression levels in 120 HCC and paired noncancerous tissues. **B** GRSF1 and YY1 expression levels were decreased in GSRF1-deficient HCC cells. **C** RIP assays showing a direct interaction between GRSF1 and YY1. UBC mRNA (which does not bind GRSF1) was used as a negative control, LMNB1 (a known RNA target of GRSF1) was used as a positive control, and GAPDH was used as a background control mRNA. **D** Silencing GRSF1 markedly shortened the half-life of YY1 mRNA in HCC cells. **E** Separation of the full-length YY1 3`UTR into different overlapping fragments. **F** Pull-down assays showing that GRSF1 specifically interacts with the 3`UTR-2205 of YY1. **G** Pull-down assays showing that GRSF1 associates with the 3`UTR 2663-2847 of YY1. **H** Luciferase reporter assays showing that GRSF1 interacts with the YY1 3`UTR. **I** Luciferase reporter assays suggesting that GRSF1 interacts with the 3`UTR-2205 of YY. **J** Luciferase reporter assays showing that GRSF1 interacts with the 3`UTR-2663 of YY1. Values are the mean± SEM (*n*=3). *p < 0.05, **p < 0.01

To better understand the mechanism underlying the tumorigenic function of GRSF1, transcriptome sequencing was performed in MHCC-97H GRSF1-deficient cells (si-GRSF1) and control cells (si-Ctrl). Since GRSF1 promoted HCC malignant biological behavior, we focused on the downstream genes that can extensively regulate HCC phenotypes. YY1, which is pivotal for promoting hepatocarcinogenesis via a wide variety of classical pathways, was identified as one of the genes with the most dramatic expression changes (fold-change >2) (Table S11). Thus, we focused on YY1 as a downstream effector of GRSF1. qRT–PCR assays using 120 HCC clinical samples confirmed that YY1 expression was markedly higher in HCC tissues than in normal tissues (*p*<0.01, Fig. 2A). qRT–PCR and western blotting assays showed that YY1 expression was significantly decreased with knockdown of GRSF1 in MHCC-97H and Hep3B cells (*p*<0.01, Fig. 2B and S1A). GRSF1 was stably overexpressed in HCC cells via a lentiviral approach (ov-GRSF1). Similarly, GRSF1 overexpression increased YY1 expression (*p*<0.01, Fig. S1B). RIP assays revealed that YY1 mRNA was enriched in the anti-GRSF1 IP antibody-precipitated sample but not in the IgG-IP sample, suggesting that GRSF1 directly interacts with YY1 mRNA in HCC (*p*<0.01, Fig. 2C). To further confirm the interaction of GRSF1 with YY1, we measured their endogenous interaction by performing immunoprecipitation assays and found that endogenous GRSF1 interacts with endogenous YY1 in HCC cells (Fig. S1C). The binding of endogenous GRSF1 to endogenous YY1 mRNA was also confirmed by RIP analysis (Fig. S1D). We further evaluated the stability of YY1 mRNA in response to GRSF1 knockdown in HCC cells and found that the half-life of YY1 mRNA was markedly shorter in GRSF1-deficient HCC cells than in the corresponding control cells (*p*<0.05, Fig. 2D). To investigate the specific binding region of GRSF1 on YY1 mRNA, we separated the full-length YY1 3`UTR into six overlapping fragments, namely, YY1 3`UTR-1348-2204, 2205-3060, 3061-3916, 3917-4772, 4773-5627 and 5628–6481 (Fig. 2E), followed by pull-down assays. As shown in Fig. 2F, GRSF1 specifically interacted with 3`UTR-2205 (nt 2205-3060) but not the 5`UTR, CDS or other 3`UTR fragments. Subsequently, we subdivided 3’UTR-2205 into four fragments (3’UTRs 2205-2418, 2419-2662, 2663-2847 and 2848–3060) and found that GRSF1 predominantly interacted with the 3’UTR 2663-2847 region (Fig. 2G). These results were supported by luciferase reporter assays, which showed that GRSF1 interacted with the YY1 3`UTR but not YY1-5`UTR or YY1-CDS (*p*<0.01, Fig. 2H). The YY1 3`UTR was then divided into six parts as previously mentioned, and corresponding luciferase reporter plasmids were generated. The results further confirmed that GRSF1 specifically interacted with 3’UTR-2205 (nt 2205-3060), which was confirmed to bind to the GRSF1 protein (*p*<0.01, Fig. 2I). We then subdivided 3’UTR-2205 into four fragments as previously mentioned and observed that YY1 3`UTR 2663-2847 dramatically lost luciferase activity upon GRSF1 knockdown (*p*<0.01, Fig. 2J). The reporter mRNA expression levels also showed the same change, suggesting that the effect of GRSF1 is at the RNA level (*p*<0.01, Fig. S1E). Altogether, these findings demonstrated that GRSF1 promotes YY1 expression by stabilizing YY1 mRNA via direct binding with the 3`UTR 2663-2847 segment.

### YY1 is an essential downstream effector of GRSF1, and feedback promotes GRSF1 expression by binding to GRSF1 promoters

**Fig. 3:**
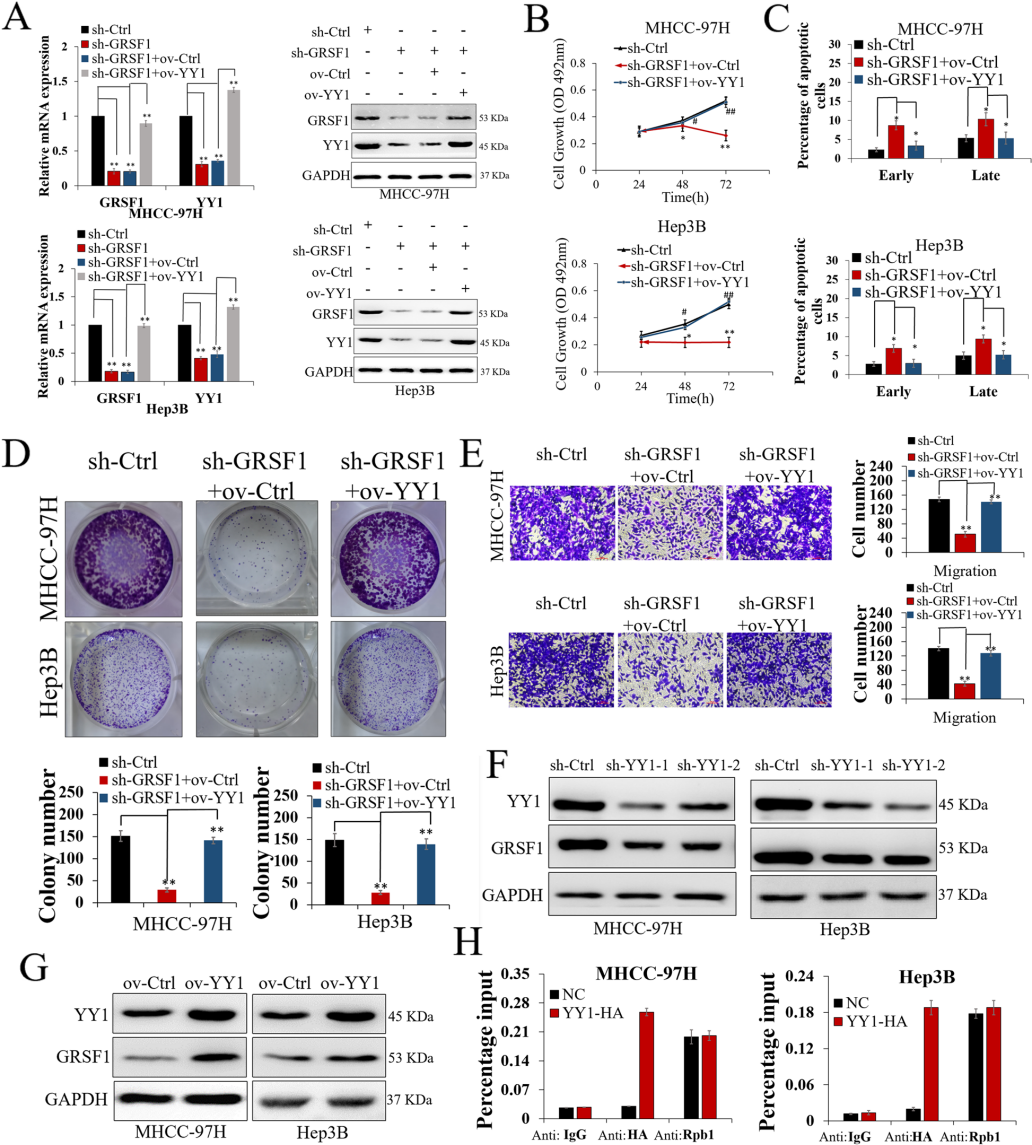
YY1 is an essential target of GRSF1, and feedback promoted GRSF1 expression. **A** GRSF1 and YY1 expression in HCC cells cotransfected with sh-GRSF1 and ov-YY1 vectors. **B-E** YY1 overexpression rescued the tumor-inhibiting effect caused by GRSF1 knockdown on HCC cell proliferation (**B**), cell apoptosis (**C**), colony formation (**D**) and migration (**E**) ability. The invaded cells in Transwell assays were quantified by counting the cells in 10 random fields (magnification, 200×). **F** GRSF1 expression levels in HCC cells were decreased following inhibition of YY1. **G** GRSF1 expression in HCC cells was increased following overexpression of YY1. **H** ChIP analysis of YY1-HA tagged fusion vector levels at the GRSF1 promoter in MHCC-97H and Hep3B cells. ChIP results were analyzed via qRT–PCR using GRSF1 promoter-specific primers and are expressed as the percentage of the input. Rpb1 was used as a positive control for GRSF1 ChIP. IgG was used as a negative control. Values are the mean± SEM (*n *= 3). *p < 0.05, **p < 0.01

To identify whether YY1 is essential for maintaining the function of GRSF1 in HCC, we performed a rescue assay. YY1 was stably overexpressed in GRSF1-deficient MHCC-97H and Hep3B cells through a virus transfection pathway (*p*<0.01, Fig. 3A and Fig. S2A). From a functional point of view, YY1 overexpression rescued the tumor-inhibiting effect of decreased GRSF1 on HCC cell proliferation, apoptosis, colony formation and migration (*p*<0.05, Fig. 3B-E). In addition, we unexpectedly found that GRSF1 expression in GRSF1-deficient HCC cells was concomitantly upregulated with increased YY1 (*p*<0.01, Fig. 3A). Thus, we speculated that YY1 was not only a downstream target of GRSF1 but also a feedback promoter of GRSF1. Using the UCSC Genome Browser on Human feb.2009 (GRCh37/hg19) assembly software, we found that there was a potential YY1 binding site in the promoter of GRSF1. We constructed HCC cells with stable YY1 silencing or overexpression using lentivirus vectors and found that GRSF1 mRNA expression was markedly decreased or increased upon alteration of YY1 expression (*p*<0.01, Fig. S2B). GRSF1 protein expression showed the same changes (Fig. 3F, G, *p*<0.01, Fig. S2C). Mechanistically, YY1-HA tagged fusion expression vectors were used to detect the binding of exogenous YY1 with the GRSF1 promoter via ChIP-qPCR. The vectors were successfully expressed in HCC cells (*p*<0.01, Fig. S2D and E). ChIP assays demonstrated that YY1 bound to the promoter of GRSF1 but not to the control region (*p*<0.01, Fig. 3H). ChIP analysis of endogenous YY1 levels at the GRSF1 promoter was also performed in MHCC-97H and Hep3B cells and showed that YY1 bound to the promoter of GRSF1 (*p*<0.01, Fig. S2F).

### miR-30e-5p inhibits YY1 and hepatocarcinogenesis by binding to the 3'UTR of YY1

**Fig. 4:**
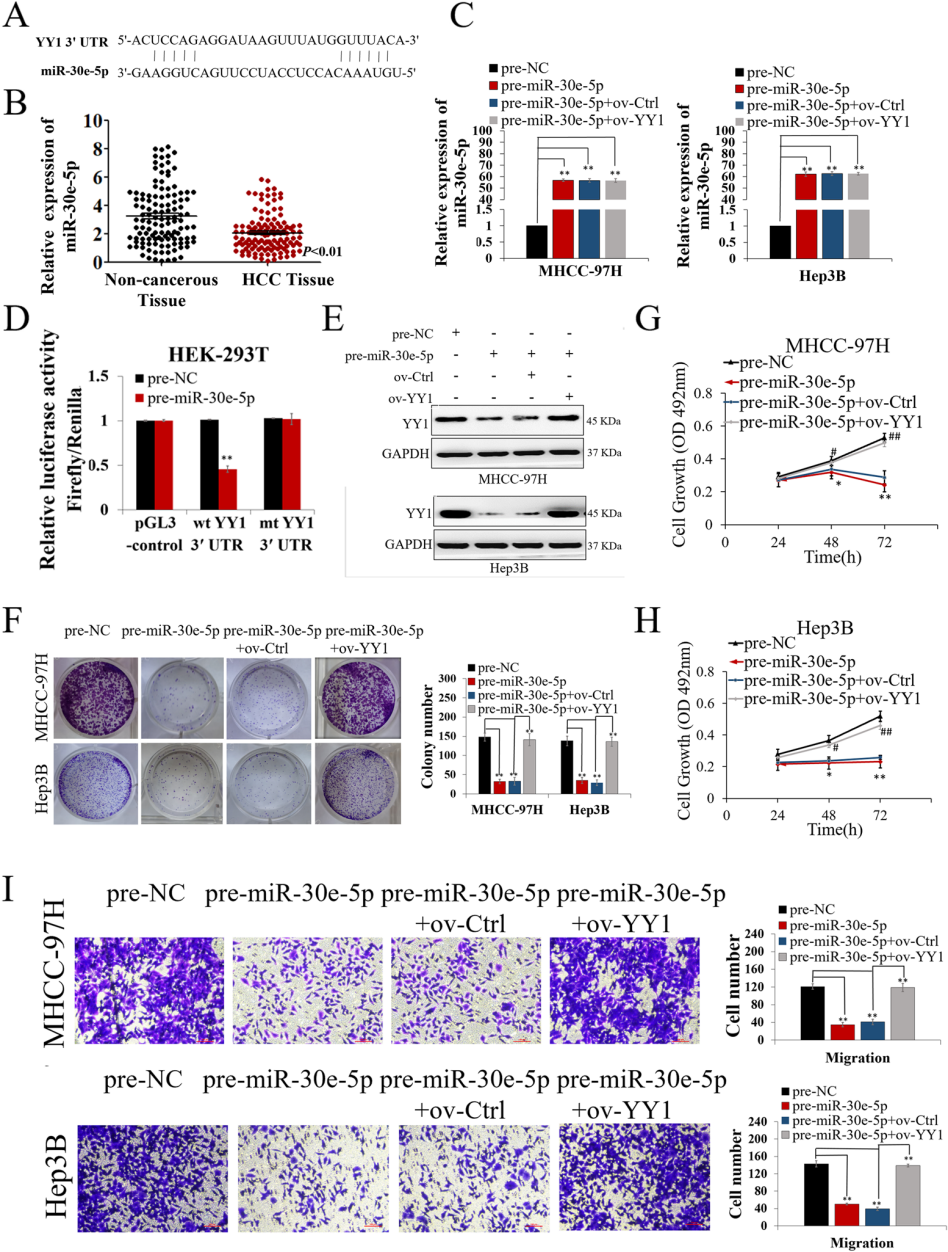
miR-30e-5p inhibited YY1 and hepatocarcinogenesis by binding to the 3'UTR of YY1. **A** miR-30e-5p and its putative binding sequence in the YY1 3'-UTR. **B** miR-30e-5p expression was decreased in HCC tissues. **C** miR-30e-5p expression in MHCC-97H and Hep3B cells was increased upon transfection with pre-miR-30e-5p but was not regulated by YY1 overexpression. **D** Luciferase reporter gene assay showing that miR-30e-5p overexpression decreased YY1 luciferase activity when combined with the wt YY1 3'-UTR. **E** YY1 expression was decreased, followed by increased miR-30e-5p expression, and upregulated upon transfection with the ov-YY1 vector in HCC cells. **F-I** Increased miR-30e-5p expression suppressed the colony formation (**F**), proliferation (**G, H**) and migration (**I**) ability of HCC cells, and the antitumor function of miR-30e-5p was counteracted by YY1 overexpression. Values are the mean± SEM (*n *= 3). *p < 0.05, **p < 0.01

The upstream regulatory network of YY1 is very complex and not yet clear. Therefore, we hoped to further explore the role of GRSF1 in the upstream regulatory network of YY1. Previous reports have revealed that YY1 is regulated by microRNAs; thus, we attempted to clarify whether there is competition between GRSF1 and microRNAs during the YY1 regulation process. We accessed the public database TargetScan (http://www.targetscan.org/) to search for miRNAs that could potentially interact with the YY1 3’UTR 2663-2847 and found that the complementary sequence of miR-30e-5p was present in this region (Fig. 4A). The role of miR-30e-5p in HCC has rarely been reported. Therefore, we performed qRT–PCR assays to evaluate miR-30e-5p expression in 120 HCC samples. The results showed that miR-30e-5p expression was lower in HCC tissues than in noncancerous tissues (*p*<0.01, Fig. 4B). Transfection with the miR-30e-5p precursor (pre-miR-30e-5p) markedly increased miR-30e-5p expression in HCC cells (*p*<0.01, Fig. 4C). A luciferase reporter assay showed that increased miR-30e-5p expression reduced the luciferase activity of YY1 with a wt YY1 3'-UTR, demonstrating that miR-30e-5p directly bound to YY1 (*p*<0.01, Fig. 4D). YY1 mRNA (*p*<0.01, Fig. S3A) and protein expression (*p*<0.05, Fig. 4E and S3B) levels were downregulated upon transfection with pre-miR-30e-5p. We upregulated YY1 expression in miR-30e-5p-overexpressing HCC cells through a virus transfection pathway (Fig. 4C and E). Functionally, increased miR-30e-5p suppressed HCC cell colony formation, proliferation and migration, while YY1 overexpression counteracted the tumor-inhibiting effect induced by pre-miR-30e-5p (*p*<0.01, Fig. 4F-I). These findings demonstrate that miR-30e-5p acts as a tumor suppressor by directly inhibiting YY1 and that YY1 can counteract the functional effects of miR-30e-5p in hepatocarcinogenesis.

### GRSF1 and miR-30e-5p competitively regulate YY1 by binding to its 3`UTR

**Fig. 5:**
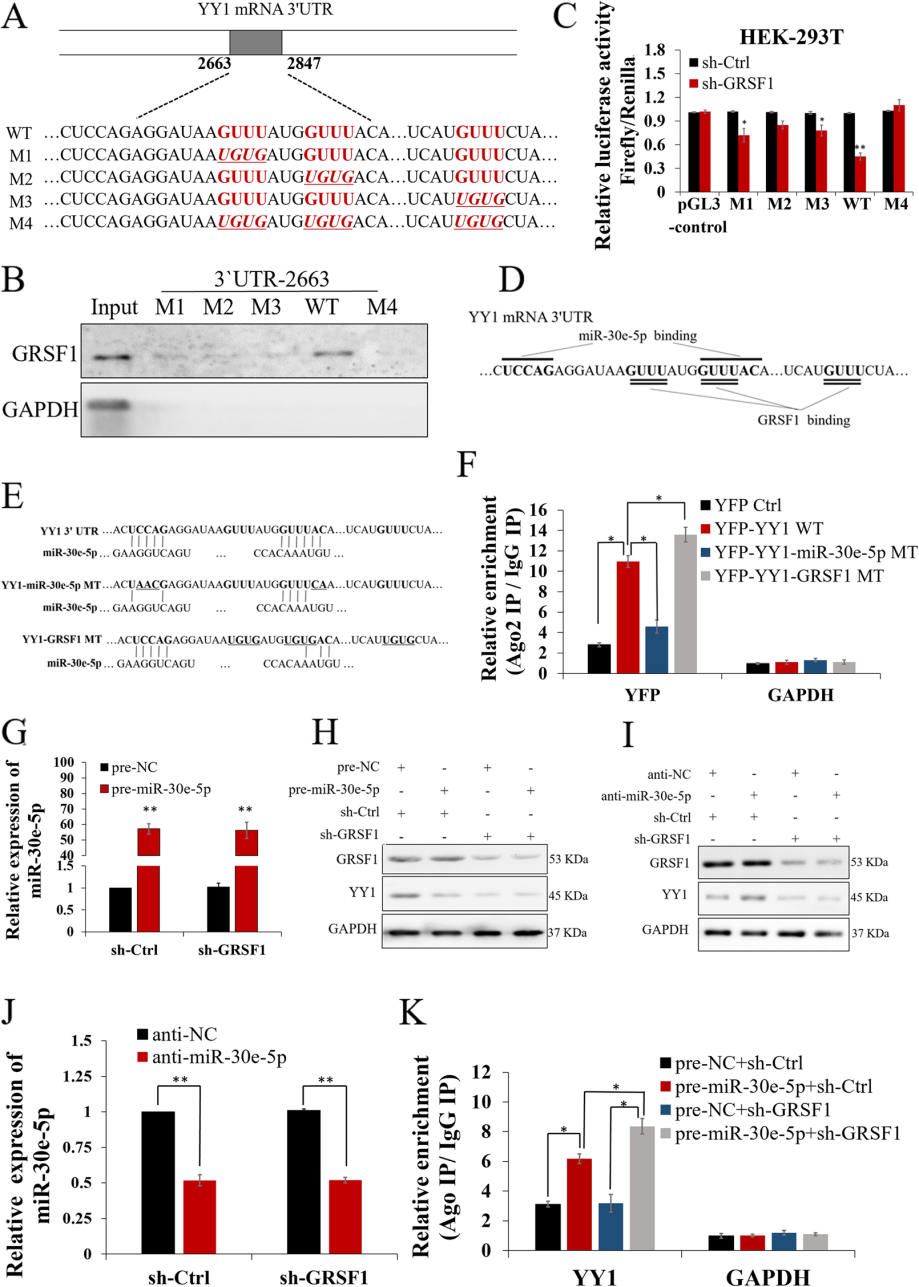
GRSF1 and miR-30e-5p competitively regulate YY1. **A, B** Wild-type (WT) fragments of YY1 3`UTR 2663-2847 and four mutants were constructed and used in RNA pull-down assays. **C** Luciferase reporter assays showed that the M1, M2 and M3 mutants partially lost the capacity to associate with GRSF1, while the M4 mutant essentially lost all ability to interact with GRSF1. **D** The binding site of YY1 for miR-30e-5p partially overlaps with GRSF1 binding sites. **E** YFP reporter construct containing only YFP (YFP Ctrl), wild-type YY1 3`UTR (YFP-YY1 WT), a mutated miR-30e-5p-binding site (YFP-YY1-miR-30e-5p MT) or a mutated GRSF1-binding site (YFP-YY1-GRSF1 MT). The underlined sequences are the mutated sites. **F** The AGO2 IP/IgG IP ratio of YY1 was increased in the YFP-YY1 WT group compared to the YFP-YY1-miR-30e-5p MT group. YFP-YY1-miR-30e-5p MT disrupts binding with miR-30e-5p, resulting in decreased RNA compared to YFP-YY1 WT. However, YFP-YY1-GRSF1 MT resulted in more enrichment in the AGO2/IP fraction than YFP-YY1 WT, suggesting that miR-30e-5p bound to the YY1 3`UTR 2663-2847 region and that the binding function was impaired by transfection with YFP-YY1-miR-30e-5p MT. GAPDH was used as a negative control. **G** miR-30e-5p expression in MHCC-97H cells was not affected by GRSF1 knockdown. **H** Western blotting assays showing that miR-30e-5p inhibits YY1 expression**. I** Western blotting assays showing that anti-miR-30e-5p promotes YY1 expression, while the promotion function was disrupted by GRSF1 knockdown. **J** miR-30e-5p expression in MHCC-97H cells was decreased by transfection with anti-miR-30e-5p but not further inhibited by GRSF1 knockdown. **K** pre-miR-30e-5p enhanced the AGO2 IP/IgG IP ratio of YY1, which was further enhanced by GRSF1 knockdown. GAPDH was used as a negative control. Values are the mean± SEM (*n *= 3). *p < 0.05, **p < 0.01

We further sought to identify the essential motifs that interacted with GRSF1 and found that the 3`UTR 2663-2847 region contains three GUUU motifs that frequently appear in the 3`UTR of YY1 mRNA. Thus, four mutants were constructed, M1, M2 and M3, which were mutated in the first, second and third GUUU motifs (GUUU to UGUG), respectively, and M4, in which all three GUUU motifs were replaced by UGUG (Fig. 5A). Subsequently, a biotin-mediated RNA pull-down assay was performed to determine the ability of the wild-type (WT) fragment and the four mutants to interact with GRSF1. The results showed that the M1, M2 and M3 mutants partially lost the capacity to associate with GRSF1, while the M4 mutant essentially lost all ability to interact with GRSF1 (Fig. 5B). Luciferase reporter assays showed similar results (Fig. 5C and Fig. S3C-E). These findings indicate that the GUUU mutation blocked GRSF1 binding to the YY1 3`UTR, demonstrating that the GUUU motifs in the YY1 3`UTR 2663-2847 are GRSF1 binding motifs. The binding site of YY1 for miR-30e-5p partially overlapped with GRSF1 binding sites (Fig. 5D), and our results showed that miR-30e-5p and GRSF1 are antagonistic in the regulation of YY1. Thus, we prepared an YFP reporter expressing a chimeric RNA construct containing the sequence of YFP, wild-type (YFP-YY1 WT) or mutated YY1 3`UTR to help better understand the coregulation of YY1 mRNA by GRSF1 and miR-30e-5p. The construct containing only the YFP sequence was identified as YFP Ctrl. YFP-YY1-miR-30e-5p MT contained mutations within the binding site of YY1 for miR-30e-5p. YFP-YY1-GRSF1 MT contained mutations that replaced the GUUU motifs in YY1 3`UTR 2663-2847 with UGUG (Fig. 5E).

After transfection of MHCC-97H cells with the sh-GRSF1 vector for 24 h, YFP reporters were applied, and then, we conducted RNA IP assays using Ago2 to identify the YFP constructs involved in the RNA-induced silencing complex (RISC). If miR-30e-5p binds to the YFP-YY1 3’ UTR, we would expect to pull down the YFP construct. The results showed that YFP-YY1-miR-30e-5p MT disrupts binding with miR-30e-5p, resulting in decreased RNA compared to YFP-YY1 WT. However, YFP-YY1-GRSF1 MT resulted in increased RNA compared to YFP-YY1 WT, suggesting that miR-30e-5p bound to the YY1 3`UTR 2663-2847 region and that the binding function was impaired by transfection with YFP-YY1-miR-30e-5p MT. The mutation induced by transfection with YFP-YY1-GRSF1 MT enhanced the interaction between miR-30e-5p and YY1 instead of disrupting their association, suggesting that GRSF1 can antagonize miR-30e-5p binding with YY1 and that mutation of GRSF1 cannot prevent miR-30e-5p binding to the YY1 3`UTR (Fig. 5F). Thus, our data showed that GRSF1 and miR-30e-5p competitively regulate YY1 expression because their binding sites overlap. We further measured the function of GRSF1 knockdown in MHCC-97H cells overexpressing miR-30e-5p using qRT–PCR and found that GRSF1 knockdown did not affect miR-30e-5p expression (*p*<0.01, Fig. 5G). Western blotting assays showed that miR-30e-5p inhibited YY1 expression (*p*<0.05, Fig. 5H, Fig. S3F). Further assays showed that transfection with anti-miR-30e-5p increased YY1 protein expression in MHCC-97H cells, while GRSF1 silencing disrupted the promotion of YY1 expression caused by anti-miR-30e-5p (*p*<0.05, Fig. 5I, Fig. S3F). Subsequently, qRT–PCR assays demonstrated that anti-miR-30e-5p successfully decreased miR-30e-5p expression, while silencing GRSF1 did not lead to a change in miR-30e-5p expression (*p*<0.01, Fig. 5J). RIP assays using an anti-Ago2 antibody demonstrated that miR-30e-5p overexpression enhanced the interaction between Ago2 and YY1 mRNA, and this interaction was further increased by GRSF1 knockdown (*p*<0.05, Fig. 5K). RIP analysis showed that overexpression of pre-miR-30e-5p reduced the binding of GSRF1 to YY1 (*p*<0.05, Fig. S3G and H). These results further suggested a competitive effect of GRSF1 and miR-30e-5p on YY1 expression.

**Fig. 6:**
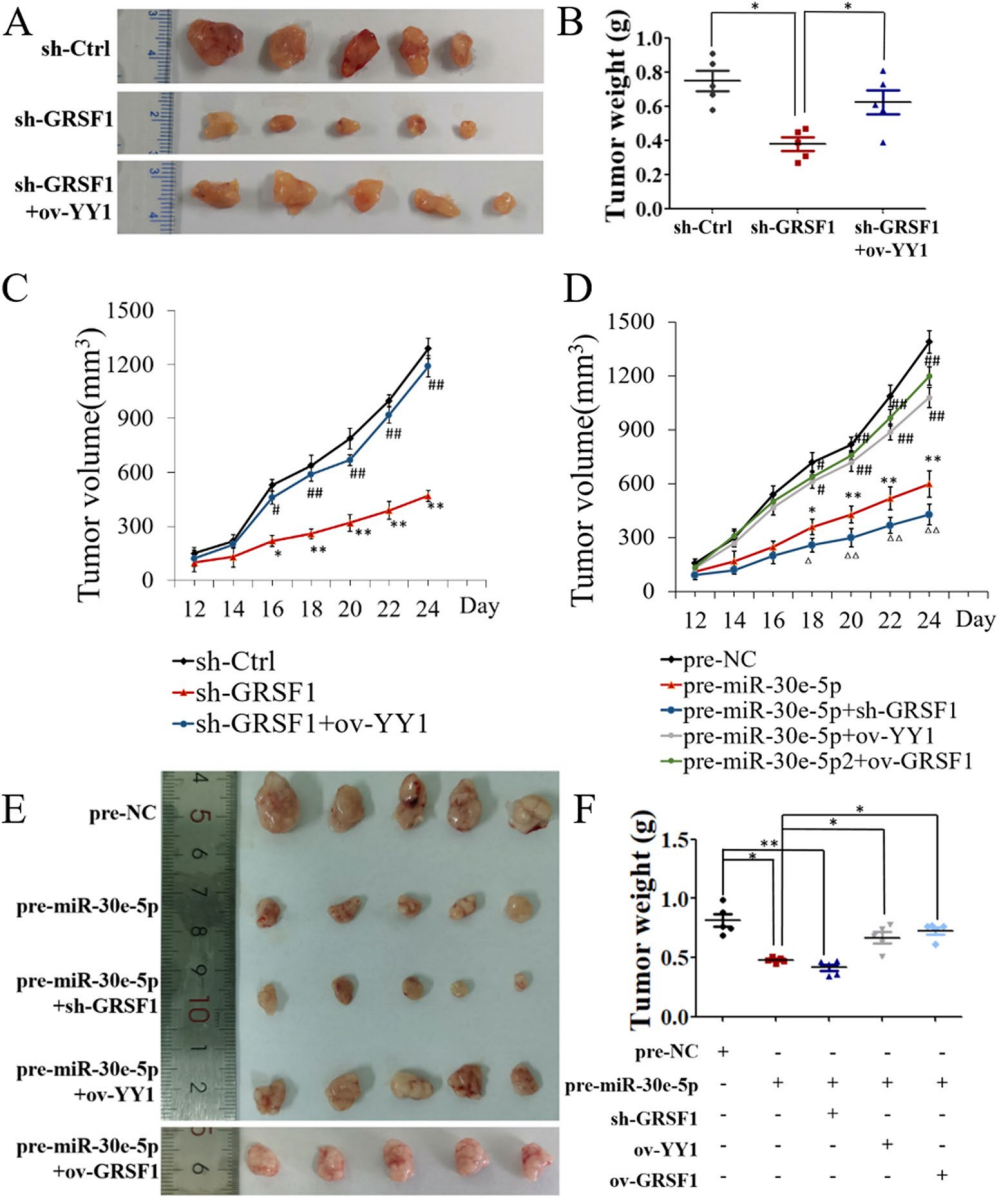
In vivo results. **A-C** Decreased GRSF1 expression suppressed HCC xenograft tumor growth (*p<0.05, **p<0.01), while YY1 overexpression counteracted the inhibitory effect of GRSF1 knockdown (^#^*p*<0.05, ^##^*p*<0.01). **D-F** miR-30e-5p overexpression suppressed HCC xenograft tumor growth (*p<0.05, **p<0.01), YY1 overexpression or GRSF1 overexpression attenuated the inhibitory effect induced by pre-miR-30e-5p (^#^*p*<0.05, ^##^*p*<0.01), and cotransfection with pre-miR-30e-5p and sh-GRSF1 resulted in even smaller tumor sizes than transfection with pre-miR-30e-5p alone (^△^*p*<0.05, ^△△^*p*<0.01). Values are the mean± SEM (*n*=5)

In addition, a xenograft model in immunodeficient mice showed that tumors derived from MHCC-97H cells with decreased GRSF1 expression resulted in smaller tumor sizes (sh-GRSF1 group) and that YY1 overexpression (sh-GRSF1+ov-YY1 group) promoted tumor growth (*p*<0.05; Fig. 6A-C). Xenograft tumors derived from MHCC-97H cells with increased miR-30e-5p expression resulted in smaller tumor sizes (pre-miR-30e-5p group) than those formed by injection with the control cells (pre-NC group). YY1 overexpression (pre-miR-30e-5p+ov-YY1 group) attenuated the inhibition of tumor growth caused by pre-miR-30e-5p. In addition, xenograft tumors derived from MHCC-97H cells cotransfected with pre-miR-30e-5p and sh-GRSF1 exhibited even smaller tumor sizes (pre-miR-30e-5p+sh-GRSF1 group) than those derived from cells transfected with pre-miR-30e-5p alone (pre-miR-30e-5p group). Xenograft tumors derived from MHCC-97H cells cotransfected with pre-miR-30e-5p and over-GRSF1 exhibited larger tumor sizes (pre-miR-30e-5p+ov-GRSF1 group) than those in the premiR-3pe-5p group (*p*<0.05; Fig. 6D-F), supporting our in vitro results. Immunohistochemistry assays of GRSF1, YY1 and Ki67 expression confirmed that GRSF1 promoted hepatocarcinogenesis upon exposure to YY1 and that YY1 feedback promoted GRSF1 expression (Supplementary material, Fig. S4A, B).

### VE821 inhibits HCC by repressing the GRSF1/YY1 pathway

**Fig. 7:**
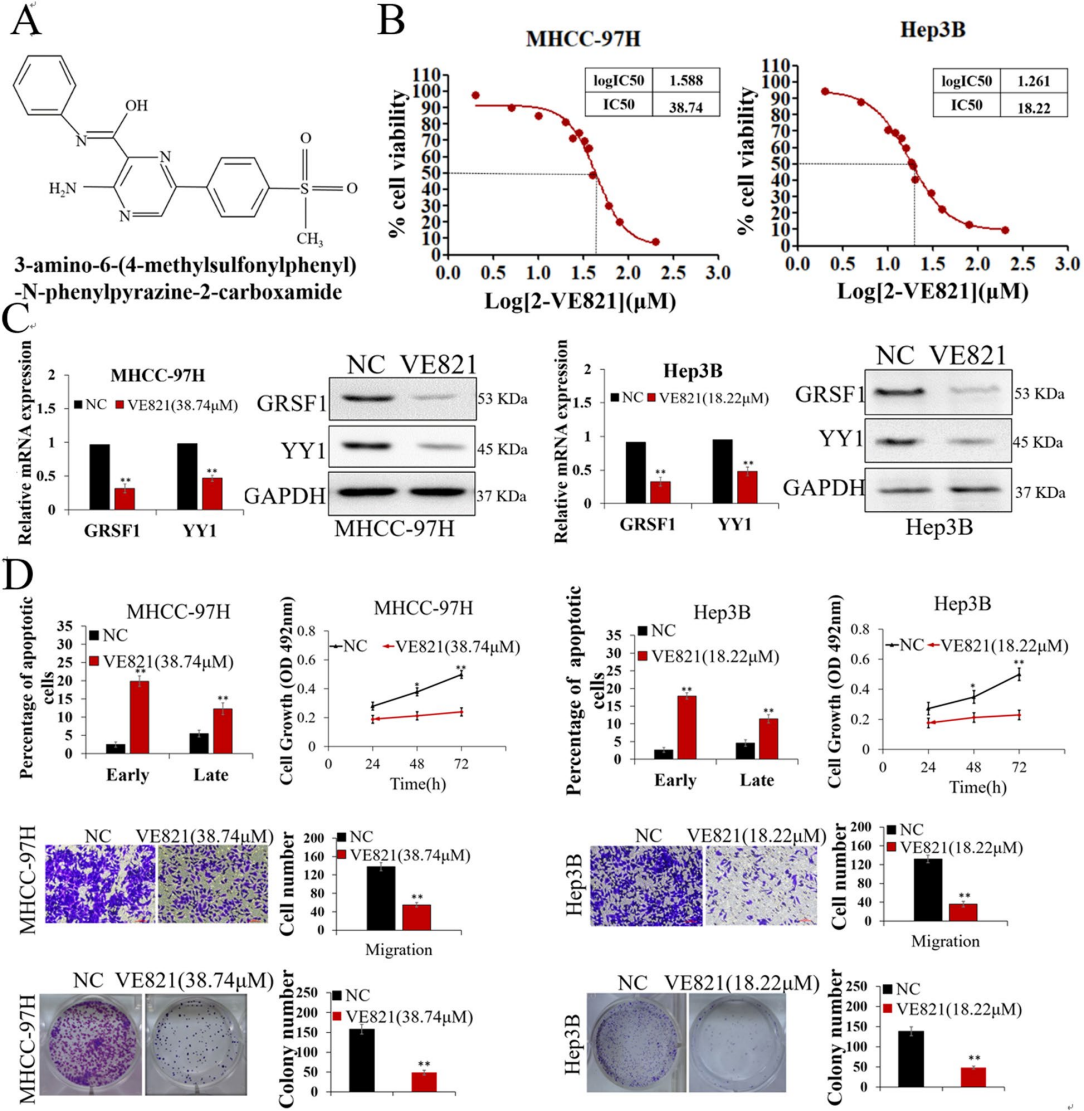
VE821 inhibits HCC by repressing the GRSF1/YY1 pathway. **A** Chemical structure of VE821. **B** Cytotoxicity analysis of VE821 in MHCC-97H and Hep3B cells. **C** GRSF1 and YY1 expression levels were decreased in MHCC-97H and Hep3B cells treated with VE821. **D** VE821 markedly inhibited the proliferation, migration, and colony formation ability of MHCC-97H and Hep3B cells and enhanced apoptosis (*n*=5). Values are the mean± SEM (*n*=3); *p<0.05, **p<0.01

**Fig. 8:**
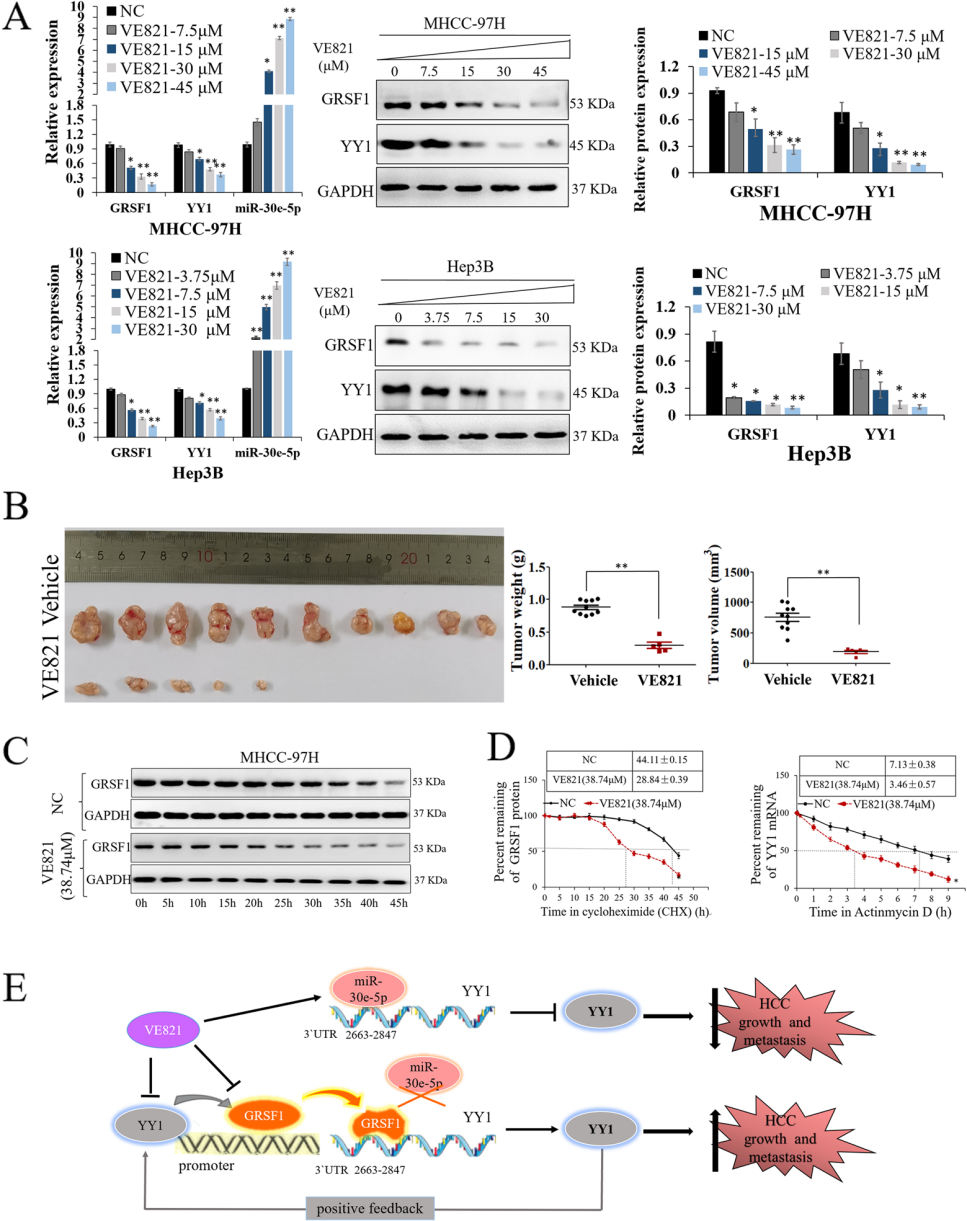
VE821 inhibits HCC by repressing the GRSF1/YY1 pathway. **A** GRSF1, YY1 and miR-30e-5p expression in MHCC-97H cells treated with different concentrations of VE821. **B** Representative images of subcutaneous tumors resected from mice in the VE821 or vehicle group. **C** CHX chase assay showing that VE821 impairs the protein stability of GRSF1. **D** VE821 shortened the YY1 mRNA half-life. **E** Proposed model for the effect of GRSF1 on hepatocarcinogenesis. GRSF1 facilitates HCC growth and metastasis by competing with miR-30e-5p for binding to the YY1 3`UTR 2663-2847, and YY1 feedback promotes GRSF1 expression by binding to GRSF1 promoters. VE821 may serve as a novel agent with potential for HCC treatment through inhibition of the GRSF1/YY1 axis. Values are the mean± SEM (*n*=3). *p<0.05, **p<0.01

Our results showed that GRSF1 promoted hepatic tumorigenesis both in vitro and in vivo, indicating that GRSF1 is a novel driver of HCC. Furthermore, downregulation of GRSF1 led to inhibition of HCC growth. These findings provide a new molecular mechanism of hepatocarcinogenesis and suggest that the GRSF1 signaling pathway may be an attractive target for therapeutic applications. Among twelve small-molecule compounds that may inhibit the growth of HCC cells screened using high-throughput screening technology, VE821 (Fig. 7A), a selective inhibitor of ataxia-telangiectasia-mutated and rad3-related protein (ATR), was found to reduce HCC cell proliferation, although its function in HCC has not been reported in detail. Our data showed that the half maximal inhibitory concentration (IC50) of VE821 was 38.74 μM in MHCC-97H cells and 18.22 μM in Hep3B cells (Fig. 7B). Furthermore, VE821 markedly suppressed GRSF1/YY1 expression in HCC cells (Fig. 7C and Fig. S4C). VE821 markedly enhanced cell apoptosis and inhibited HCC cell proliferation, migration and colony formation (*p*<0.05, Fig. 7D). In addition, VE821 inhibited GRSF1 and YY1 expression and promoted miR-30e-5p expression in a dose-dependent manner in HCC cells (*p*<0.01, Fig. 8A).

Immunodeficient mice with xenografts derived from MHCC-97H cells were randomly given intraperitoneal injections of VE821 or vehicle. The results revealed that mice treated with VE821 exhibited smaller tumor volumes and lighter tumor weights (*p*<0.05, Fig.8B). Immunohistochemistry assays showed that GRSF1, YY1 and Ki67 expression was decreased in the VE821 group tumor tissue (*p*<0.05; Fig. S4D-E). qRT–PCR assays demonstrated that GRSF1, YY1 and Ki67 expression was decreased in the VE821 group xenograft tumor tissue, while miR-30e-5p expression was increased (*p*<0.01; Fig. S4F). The results verified that VE821 prevented HCC progression by inhibiting the GRSF1/YY1 signaling pathway. Further in vivo experiments showed that VE821 had reasonable biological safety in animal models (Supplementary material, Fig. S5, Table. S4-10).

Next, we performed protein degradation analysis in MHCC-97H cells treated with VE821 or not and found that VE821 obviously impaired the protein stability of GRSF1 (*p*<0.05, Fig. 8C). Moreover, RNA stability measurements demonstrated that MHCC-97H cells treated with VE821 exhibited an obviously shorter YY1 mRNA half-life than those not treated with VE821 (NC) (*p*<0.05, Fig. 8D), suggesting that VE821 inhibited the stability of YY1 mRNA by reducing the stability of the GRSF1 protein.

To further demonstrate that VE821 functions by inhibiting GRSF1, we treated GRSF1-deficient MHCC-97H and Hep3B cells with VE821 and found that VE821 could not further reduce GRSF1-deficient HCC cell proliferation, colony formation, migration (*p*<0.05; Fig. S6A-D) and tumor growth (*p*<0.05; Fig. S7) in the background of GRSF1 depletion. In short, these results demonstrated that VE821 prevented HCC progression by suppressing the GRSF1/YY1 pathway, providing a novel option for HCC treatment.

## Discussion

In this study, we discovered the oncogenic function of the RBP GRSF1 in HCC and the mutual regulatory network among GRSF1, YY1 and miR-30e-5p, providing novel mechanistic insight into the pathogenesis of HCC.

The results demonstrated that GRSF1 expression was frequently upregulated in HCC tissues and cells. Similar to our results, GRSF1 has been found to be upregulated in cervical cancer and involved in oncogenic activity by regulating miR-G-10 [11] or MIR-G-1 [12]. In addition, GRSF1 participates in the miR-346-mediated promotion of the malignant phenotype of cervical cancer cells by enhancing AGO2 [26]. Elucidation of the interaction networks between RBPs and cancer-related RNA targets has provided new avenues for the pathogenesis of HCC and attracted considerable attention. A number of RBPs have a significant impact on HCC tumorigenesis by regulating numerous mRNAs at the translational or posttranslational level. SORBS2 was shown to inhibit hepatocarcinogenesis by stabilizing RORA expression [27]. RDM1 suppressed HCC cell proliferation by targeting p53 [28]. Here, we uncovered that the RBP GRSF1 promoted YY1 expression by enhancing its mRNA stabilization. Previous studies showed that RBPs usually bind to specific sequence regions of target mRNAs. HuR has been shown to bind with AU-rich elements (AREs) of CMTM6 [12]. RBM38 enhanced PTEN mRNA stabilization by binding to multiple AU/U elements in the 3`UTR of PTEN [29]. In our study, by separating the YY1 3`UTR into different overlapping fragments, constructing mutants containing different motifs and subsequently performing pull-down assays and luciferase assays, we found that the GUUU motifs in YY1 3`UTR 2663-2847 formed a specific binding region for GRSF1. In addition, the results reported here indicate that YY1 promotes hepatocarcinogenesis, which is consistent with previous reports. YY1 was confirmed to promote HCC progression by activating lncMER52A [30]. YY1 promoted HCC cell growth by facilitating linc01134/miR-324-5p/IGF2BP1 [31]. Similarly, the role of YY1 in facilitating HCC tumorigenesis via the elimination of fatty acid oxidation has also been validated [32]. Our data not only showed that GRSF1 promoted HCC via posttranscriptional regulation of YY1 but also suggested the interesting role of the GRSF1/YY1 positive feedback loop in HCC pathogenesis.

RBPs and miRNAs are two common approaches to 3′UTR-dependent gene regulation. Numerous independent studies have shown that RBPs and miRNAs can coregulate target gene mRNAs. PUM2 represses osteosarcoma by competitively binding to the STARD13 3'UTR against miR-590-3p and miR-9 [33]. Similarly, transformer 2β binds to BCL2 mRNA by antagonizing the binding of miR-548-3p [34]. HuR promotes lung cancer by opposing miR-873 and miR-125a-3p, competitively binding to CDK3 mRNA [35]. Similar findings in another study showed that HuR and miR-494 functionally compete for binding with the nucleolin 3'UTR in cervical carcinoma [36]. These results verify that RBPs can regulate target mRNAs via joint activity with miRNAs when binding regions for miRNAs overlap with those for RBP. Our study showed that GRSF1 interacts with one GUUU sequence within the miR-30e-5p binding site in the YY1 3`UTR and antagonized miR-30e-5p binding to regulate YY1 expression. Thus, we discovered novel crosstalk between GRSF1 and miR-30e-5p during HCC tumorigenesis. We note that the regulatory mechanism underlying the abnormal expression of YY1 in HCC is very likely more complicated and not limited to coregulation between GRSF1 and miR-30e-5p. It should also be mentioned that YY1 is not the sole target of GRSF1 and miR-30e-5p. Several other targets of GRSF1 have been identified, including mTOR, miR-G, TMED5 and LMNB1 [14–16]. In addition, USP22, EGFR, MET and MAPK were reported to act as targets of miR-30e-5p in human cancers [24, 25, 37]. Whether these targets are also coregulated by GRSF1 and miR-30e-5 remains to be determined. Among these targets, whether YY1 is the most important determinant of the activities of GRSF1 and miR-30e-5p remains unclear. These issues are limitations of our study. However, the competitive regulation between GRSF1 and miR-30e-5p uncovered in the present study could provide novel insight into the regulatory mechanism of YY1 in HCC.

Recently, although promising emerging treatment options have expanded the pool of potential HCC systemic treatments, the clinical prognosis remains disturbingly poor [38]. Hence, further research directed toward developing additional approaches to treat HCC remains urgent. In this study, we identified that VE-821 inhibits HCC growth in vitro and in vivo. Our results are in accordance with the antitumor agent role of VE821 demonstrated in other studies. Dias et al. reported that VE-821 can induce cancer cell death [39]. Another report demonstrated that VE-821 induces BRCA1 mutant ovarian cell death [40]. The data in the present study revealed that VE-821 prevents HCC progression by repressing the GRSF1/YY1 axis and enhancing miR-30e-5p expression. Whether VE-821 can be used as a novel clinical treatment option for HCC patients deserves deeper study.

## Conclusions

Overall, this study investigated the expression patterns and functions of GRSF1, YY1, and miR-30e-5p in HCC. We revealed that GRSF1 functions as a novel oncogenic RBP in HCC by stabilizing oncogenic YY1 mRNA. YY1 feedback promoted GRSF1 expression by binding to GRSF1 promoters. GRSF1 and miR-30e-5p competed to regulate YY1 by binding to its 3`UTR 2663-2847 region. Furthermore, we discovered that VE-821 could block HCC tumorigenesis by inhibiting the GRSF1/YY1 axis. Our data provide novel mechanistic insight into the pathogenesis of HCC and identify an additional promising therapeutic target for HCC.

## Supplementary Information

**Additional file 1.**

**Additional file 2.**

## Data Availability

The datasets supporting the conclusions of this article are included within the article and its Additional files. The raw data used for Transcriptome sequencing assays have been deposited in Genome Sequence Archive (https://ngdc.cncb.ac.cn/gsa-human/) in National Genomics Data Center under accession codes HRA001615.

## Abbreviations

RBPS: RNA Binding Protein
GRSF1: G-rich sequence binding factor 1
YY1: Yin-Yang 1
HCC: Hepatocellular Carcinoma
OS: Overall Survival
DFS: Disease free survival
VE821: 3-amino-6-(4-methylsulfonylphenyl)-N-phenylpyrazine-2-carboxamid
