# Correction: The RNA-binding protein GRSF1 promotes hepatocarcinogenesis via competitively binding to YY1 mRNA with miR-30e-5p

**DOI:** 10.1186/s13046-022-02392-4

**Published:** 2022-05-25

**Authors:** Lili Han, Chen Huang, Xiaofei Wang, Dongdong Tong

**Affiliations:** 1 Department of Oncology, The Second Affiliated Hospital, College of Medicine, Xi’an Jiaotong University, Xi’an, 710004 Shaanxi China; 2 Key Laboratory of Environment and Genes Related to Diseases, Ministry of Education of China, Xi’an Jiaotong University, No.277 Yanta West Road, Xi’an, 710061 Shaanxi Province China

**Correction: J Exp Clin Cancer Res 41, 17 (2022)**

**https://doi.org/10.1186/s13046-021-02217-w**

Following publication of the original article [1], errors were identified in Figs. 4 and 7, specifically:

- Fig. 4i: incorrect transwell assay images used for pre-NC and pre-miR-30e-5p + ov-YY1 expression in MHCC-97H; the correct images are now used
- Fig. 7d: incorrect transwell assay image used for NC MHCC-97H; the correct image is now used

The corrected figures are given here. The correction does not have any effect on the final conclusions of the paper. The original article has been corrected.

**Fig. 4:**
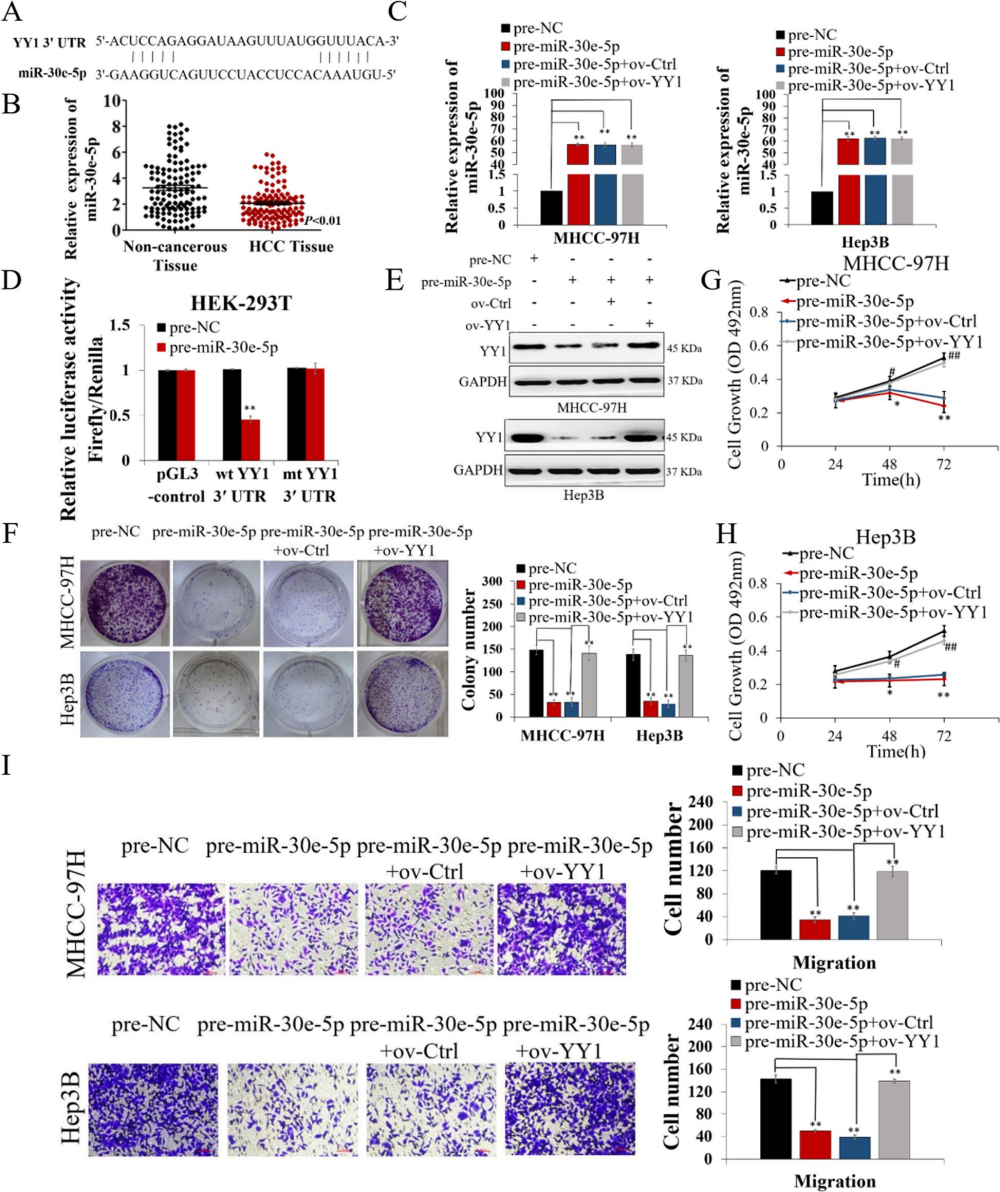
miR-30e-5p inhibited YY1 and hepatocarcinogenesis by binding to the 3’UTR of YY1. **A** miR-30e-5p and its putative binding sequence in the YY1 3′-UTR. **B** miR-30e-5p expression was decreased in HCC tissues. **C** miR-30e-5p expression in MHCC-97H and Hep3B cells was increased upon transfection with pre-miR-30e-5p but was not regulated by YY1 overexpression. **D** Luciferase reporter gene assay showing that miR-30e-5p overexpression decreased YY1 luciferase activity when combined with the wt YY1 3′-UTR. **E** YY1 expression was decreased, followed by increased miR-30e-5p expression, and upregulated upon transfection with the ov-YY1 vector in HCC cells. F-I Increased miR-30e-5p expression suppressed the colony formation (**F**), proliferation (**G, H**) and migration (**I**) ability of HCC cells, and the antitumor function of miR-30e-5p was counteracted by YY1 overexpression. Values are the mean ± SEM (*n* = 3). *p < 0.05, **p < 0.01

**Fig. 7:**
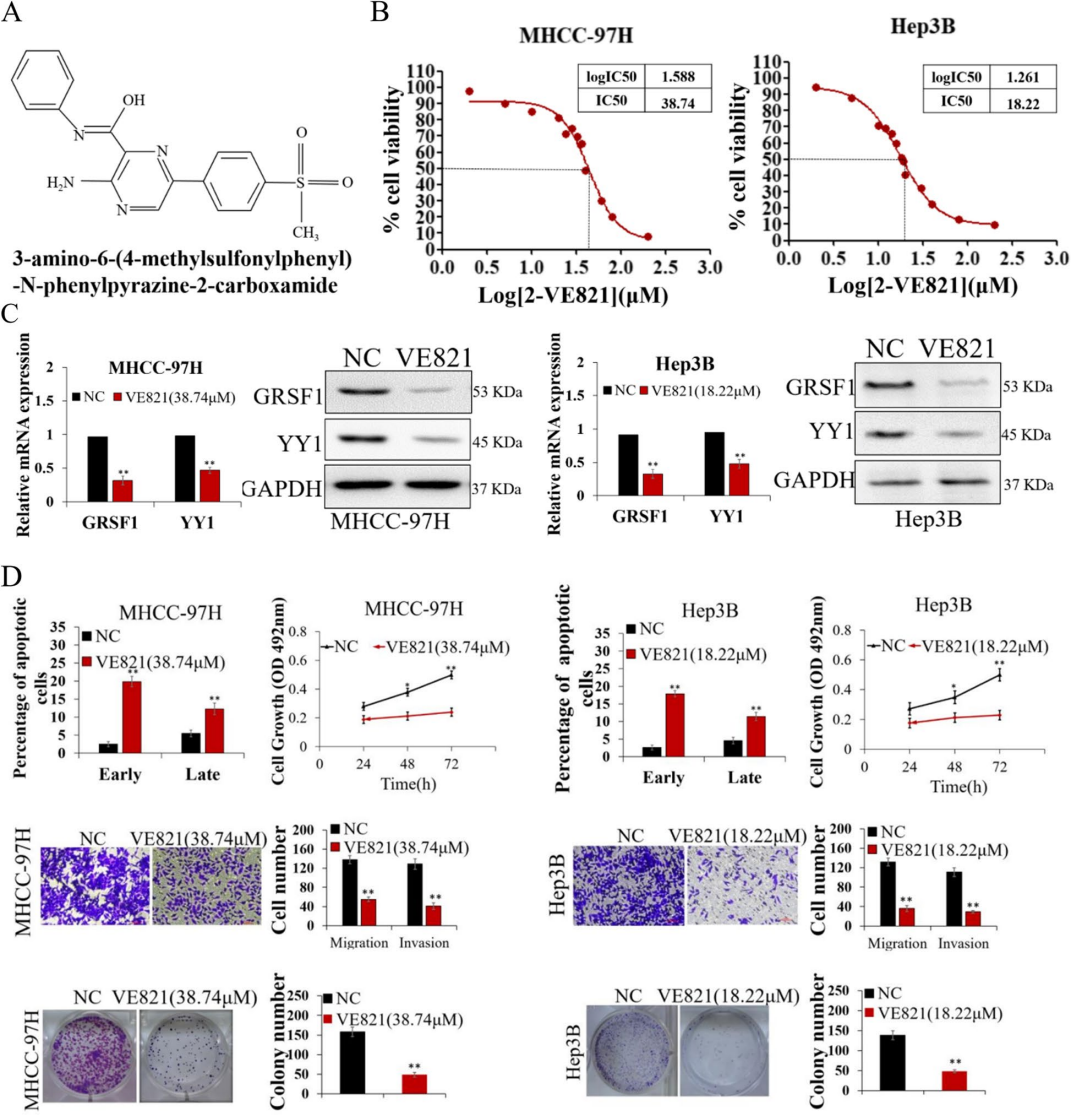
VE821 inhibits HCC by repressing the GRSF1/YY1 pathway. **A** Chemical structure of VE821. **B** Cytotoxicity analysis of VE821 in MHCC-97H and Hep3B cells. **C** GRSF1 and YY1 expression levels were decreased in MHCC-97H and Hep3B cells treated with VE821. **D** VE821 markedly inhibited the proliferation, migration, and colony formation ability of MHCC-97H and Hep3B cells and enhanced apoptosis (*n* = 5). Values are the mean ± SEM (*n* = 3); *p < 0.05, **p < 0.01
