# Supplementary material for: The RNA-binding protein GRSF1 promotes hepatocarcinogenesis via competitively binding to YY1 mRNA with miR-30e-5p
Source: J Exp Clin Cancer Res. 2022 Jan 8;41:17. doi: 10.1186/s13046-021-02217-w (PMC8742353; doi:10.1186/s13046-021-02217-w)
Supplement: Supplementary file 2 — Additional file 2. [file 13046_2021_2217_MOESM2_ESM.docx]

**Additional methodological details**

**MTT assay**

HCC cells were seeded in a 96-well plate and cultured for 24, 48 and 72 h. MTT solution was added to the cells for 4 hours, and then, the medium was replaced with fresh medium containing dimethyl sulfoxide. The absorbance was assessed at a 492 nm wavelength using a plate reader (Bio Rad Laboratories, Hercules, CA, USA).

**Colony-forming assays**

HCC cells were seeded into a 12-well plate (50 cells/well) and incubated in a humidified atmosphere for 10-12 days. The colonies were fixed with methanol, stained with crystal violet solution (Solarbio, Beijing, China) and observed under an inverted microscope (Nikon, Tokyo, Japan).

**mRNA decay assay**

HCC cells were seeded into 6-well plates and treated with actinomycin D (0.8 μg/mL, Sigma, St. MO, USA). YY1 RNA levels in HCC cells were measured via qRT–PCR at 2, 4, 6, 8, 10 and 12 h after transfection with sh-GRSF1 or the control vectors.

**Immunoprecipitation assays**

For immunoprecipitation assays to investigate the endogenous interaction between GRSF1 and YY1, endogenous GRSF1 and YY1 were separately immunoprecipitated from MHCC-97H and Hep3B cells. Cells were lysed in NP-40 cell lysis buffer. Cell lysates were incubated with Protein A/G-agarose beads (GE Healthcare) and the indicated antibodies (anti-GRSF1, anti-YY1 or IgG) overnight at 4 °C. The immunocomplexes were resolved in SDS loading buffer and analyzed via western blotting.

**Animal model assays**

Hematoxylin and eosin (HE) staining was carried out in the major organs. The tissues were fixed with 4% formaldehyde solution, embedded in paraffin, stained with hematoxylin (Servicebio, China), and treated with a hydrochloric acid alcohol solution and a weak ammonia solution (Sinopharm, Ecuador) for 20 s. Subsequently, the tissue slices were stained with eosin (Solarbio, Turkey), dehydrated, made transparent and observed under a microscope (Nikon, Japan).

**The biological safety and distribution of VE821 in animal models**

A total of 20 five-week-old male immunodeficient mice were subcutaneously inoculated with MHCC-97H cells. When the tumors grew to 100 mm^3^, the mice were randomly divided into 4 groups, namely, the VE821 1, 4, 6 and 12 h groups. VE821 (15 mg/kg) was injected. At 1, 4, 6 and 12 h after the last administration, the mice were sacrificed. The tissue samples were treated with physiological saline and homogenized. Fifty microliters of plasma or tissue homogenate was treated with 250 μL of methanol. After vortexing for 5 min, the mixture was centrifuged at 18,000 rpm for 10 min, dried with nitrogen gas, and analyzed using a liquid mass spectrometry instrument composed of a Thermo liquid system connected to a TSQ triple quadrupole mass spectrometer (Shier Technology Co., Ltd.).

To measure the LD50 of VE821 in healthy mice, a total of 15 mice were randomly divided into 3 groups. After fasting without water for 12 h, the mice were intragastrically administered vehicle or VE821 at 15 mg/kg or 10 g/kg. The diet, activity, health and death of the mice were closely observed. At 10 days after the last administration, the mice were sacrificed. HE staining assays of the major organ tissues were carried out as described above.

A total of 12 healthy mice were randomly divided into two groups and intraperitoneally injected with VE821 (15 mg/kg) or vehicle. One day after the last treatment with vehicle or VE821, routine blood and biochemical examinations were performed. HE staining assays of major organ tissues were also carried out as described above.

**Supplement results**

**GRSF1 and miR-30e-5p competitively regulate YY1 by binding to its 3`UTR**

Immunohistochemistry assays of HCC xenograft samples showed that GRSF1, YY1 and Ki67 expression levels were decreased in the sh-GRSF1 group tumor tissue but were markedly recovered in the sh-GRSF1+ov-YY1 group tumor tissue (*p*<0.05; Fig. S2G), indicating that GRSF1 promotes hepatocarcinogenesis upon exposure to YY1 and that YY1 feedback promotes GRSF1 expression. YY1 and Ki67 expression levels were decreased in the pre-miR-30e-5p group tumor tissue compared with pre-NC group tissue but were recovered in the pre-miR-30e-5p+ov-YY1 and pre-miR-30e-5p+ov-GRSF1 group tumor tissue (*p*<0.05; Fig. S2H).

**The biological safety and distribution of VE821 in animal models**

Blood biochemistry and routine examination of the immunodeficient mice showed no significant differences between the VE821 groups and vehicle group (Table. S4 and 5). In tumor-bearing immunodeficient mice, VE821 did not [lead](javascript:;) [to](javascript:;) liver damage, revealed by the levels of glutamic-pyruvic transaminase and glutamic oxalacetic transaminase; kidney damage, revealed by the levels of blood urea nitrogen and creatinine; or blood toxicity, revealed by the levels of red blood cells and hemoglobin and the white blood cell count. HE staining results suggested that VE821 treatments did not cause obvious pathological changes in the major organs (Fig. S3E). Furthermore, the distribution of VE821 in tumor-bearing immunodeficient mice was evaluated. First, the concentration of a VE821 standard solution was tested. The results presented in Table S6 suggest that the standard curve showed good linearity. Further results showed that the drug concentration in renal tissue reached its peak at 4 h post injection, while the drug concentration in other tissues was significantly decreased. At 6 h, the drug concentration in each tissue decreased further, whereas the drug concentration in tumor tissue tended to slightly increase. The high initial kidney uptake was almost completely cleared within 12 h, whereas the tumor uptake remained high or even tended to slightly increase. Other organs demonstrated low uptake at 12 h after injection (Table. S7). The results showed that VE821 was relatively selectively accumulated in tumor tissues compared with other organs in the animal models.

To further measure the general toxicity of VE821, in vivo experiments were carried out in healthy mice. Within 24 h, the mice were treated with vehicle or VE821 at 15 mg/kg or 10 g/kg. No death occurred in mice in the administration group, indicating that VE821 was well tolerated in mice. At 10 days after the last administration, the mice were sacrificed. HE staining showed that VE821 at 15 mg/kg or 10 g/kg did not result in significant toxicity to the major organs of healthy mice (Fig. S4A).

Another 10 healthy mice were randomly divided into two groups and intraperitoneally injected with VE821 (15 mg/kg) or vehicle. The blood biochemical indices indicated no significant or obvious liver or kidney damage (Table S8) or blood toxicity (Table S9) caused by VE821 treatments. HE staining further showed that VE821 treatment did not result in significant toxicity to the major organs of healthy mice (Fig. S4B). Moreover, the body weights of the healthy mice in the VE821 and vehicle groups within 14 days were [very](javascript:;) similar (Table. S10). These results confirmed the biological safety of VE821 in mice.

**Abbreviations for supplementary:**

WBC: White Blood Cell Count

RBC: Red Blood Count

HGB: Hemoglobin

MCV: Mean Corpuscular Volume

RDW: Red cell distribution width

MCH: Mean Corpuscular Hemoglobin

MCHC: Mean Corpusular Hemoglobin Concerntration

PLT: [Platelet count](javascript:;)

TP: [total](javascript:;) [protein](javascript:;)

ALB: [albumin](javascript:;)

GLB: [globulin](javascript:;)

ALT: [glutamic-pyruvic](javascript:;) [transaminase](javascript:;)

AST: [glutamic](javascript:;) [oxalacetic](javascript:;) [transaminase](javascript:;)

URE: Urea

CRE: Creatinine

UA: [Uric](javascript:;) [Acid](javascript:;)

GLU: [Glucose](javascript:;)

TC: Cholesterol

TG: [Triglyceride](javascript:;)

**Supplementary Figures**

**Fig. S1**

**
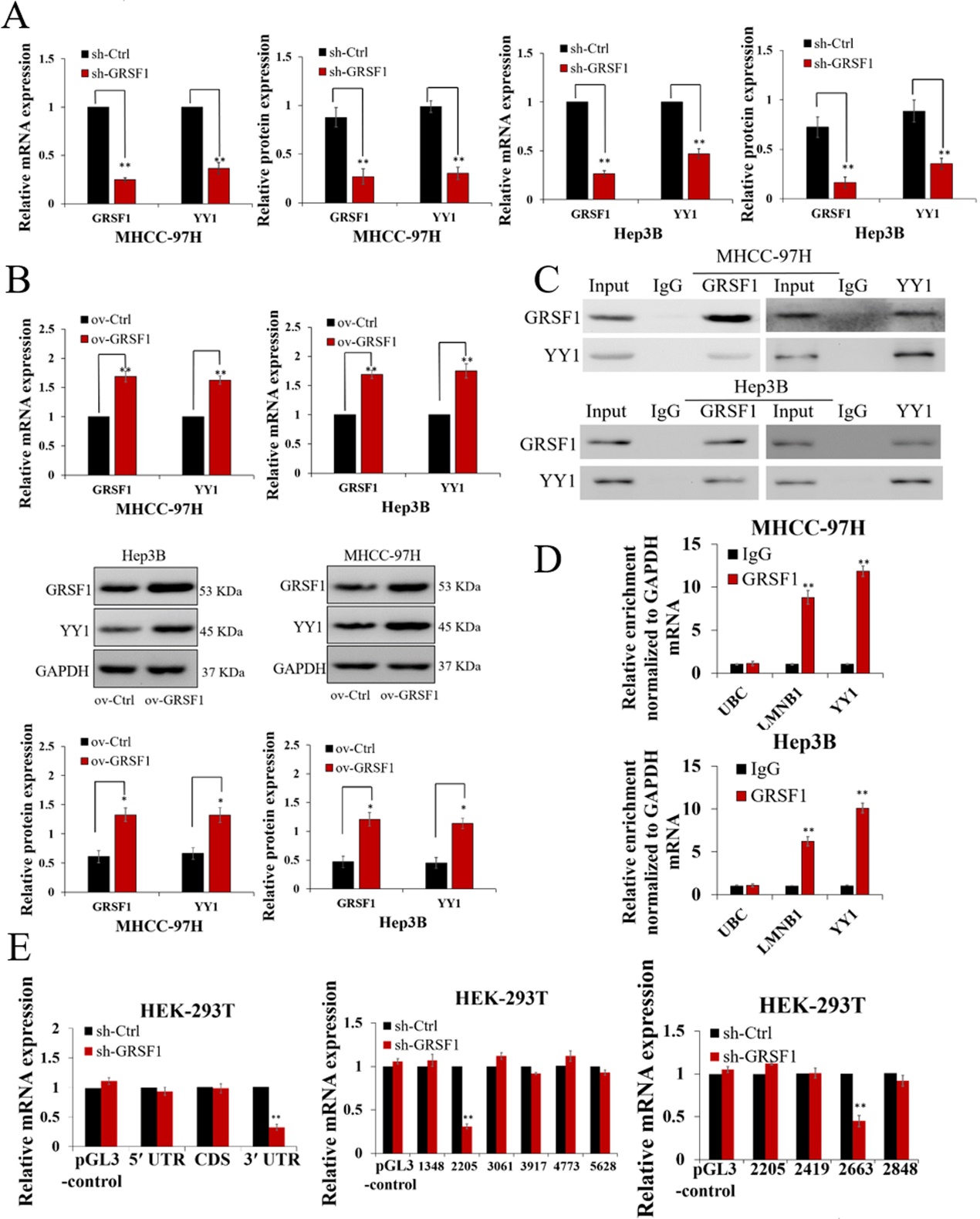
**

**Fig. S1. A.** GRSF1 and YY1 expression levels were decreased with knockdown of GRSF1. **B.** GRSF1 and YY1 expression levels were increased in GSRF1-overexpressing HCC cells. **C.** Endogenous GRSF1 interacts with endogenous YY1 in HCC cells, reflected by immunoprecipitation assay results. **D.** Binding of endogenous GRSF1 to endogenous YY1 mRNA was confirmed by RIP analysis. The abundance of UBC (nontarget RNA, negative control mRNA), LMNB1 (a known RNA target of GRSF1, positive control mRNA) and YY1 mRNA in GRSF1 IP and IgG IP control samples was assessed via RT–qPCR analysis. These results were normalized to the levels of GAPDH (control mRNA) in each sample and then plotted as the enrichment of mRNAs in the GRSF1 IP relative to the IgG IP samples. **E.** Decreased GRSF1 reduced the reporter RNA expression levels in HEK-293T cells transfected with the YY1 3`UTR but not the YY1-5`UTR or YY1-CDS. Transfection with the YY1 3`UTR 2205-3060 and 3`UTR 2663-2847 regions decreased reporter mRNA expression upon GRSF1 knockdown. **p*<0.05, ***p*<0.01.

**Fig. S2.**


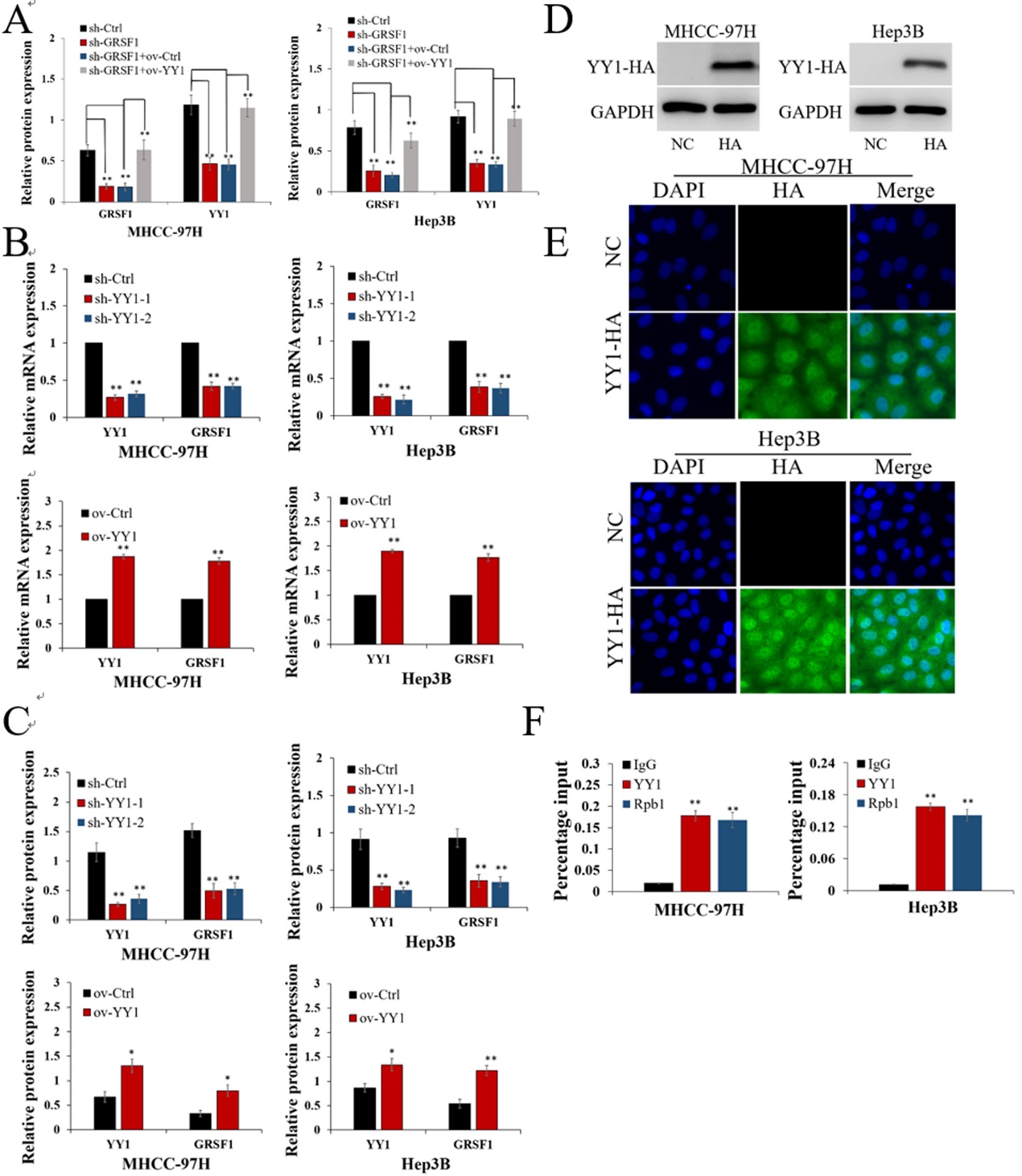


**Fig. S2. A.** Western blot analysis of GRSF1 and YY1 expression in HCC cells. **B.C.** GRSF1 expression levels were decreased following YY1 knockdown but increased upon YY1 overexpression in HCC cells. **D.** Western blot showing the expression of the YY1-HA tagged fusion expression vectors in HCC cells. **E**. Immunofluorescence analysis of the expression of YY1-HA tagged fusion vectors in HCC cells. Nuclei were stained with DAPI. **F.** ChIP analysis of endogenous YY1 levels at the GRSF1 promoter in HCC cells. Immunoprecipitated chromatin was amplified with PCR using GRSF1 promoter-specific primers, and the results are expressed as the percentage of the input. Rpb1 was used as a positive control, and IgG was used as a negative control. Values are the mean± SEM (n=3). ***p*<0.01.

**Fig. S3**

**
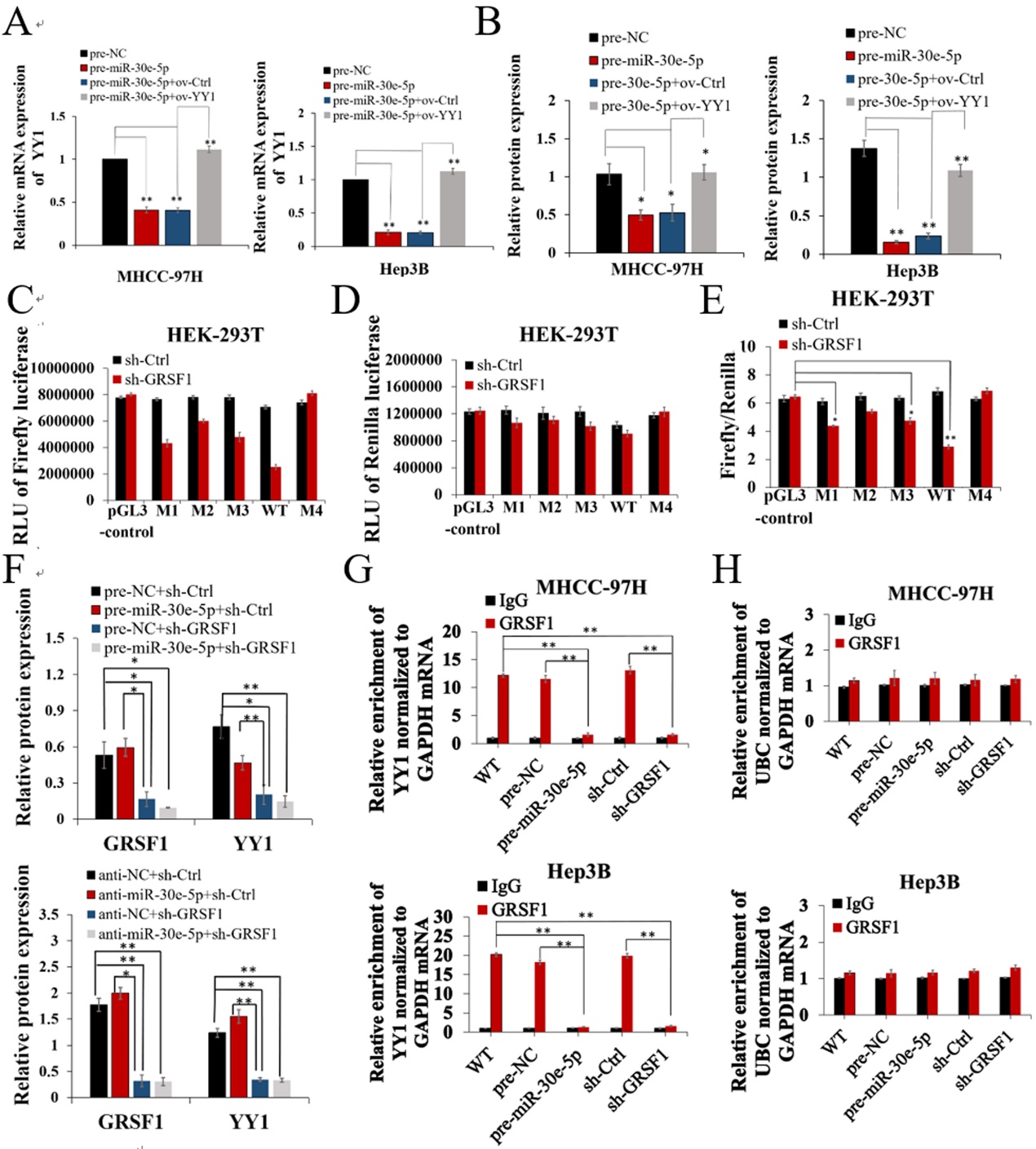
**

**Fig. S3. A.** **B.** YY1 mRNA (**A**) and protein (**B**) expression was decreased following increased miR-30e-5p expression and upregulated upon transfection with the ov-YY1 vector in HCC cells. **C.** The RLU of firefly luciferase was measured in HEK293T cells transfected with different luciferase constructs or pGL3-control. **D.** The RLU of Renilla luciferase in different groups. **E.** The ratio of firefly luciferase RLU and Renilla luciferase RLU in different groups. **F**. Western blot analysis of GRSF1 and YY1 expression in HCC cells. **G**. RIP analysis showed that the abundance of YY1 mRNA in GRSF1 IP was reduced following overexpression of pre-miR-30e-5p and inhibition of GRSF1 in MHCC-97H and Hep3B cells. **H.**UBC (nontarget RNA, negative control mRNA) mRNA levels in GRSF1 IP were similar in different groups. Values are the mean± SEM (n=3). **p*<0.05, ***p*<0.01.

**Fig. S4**


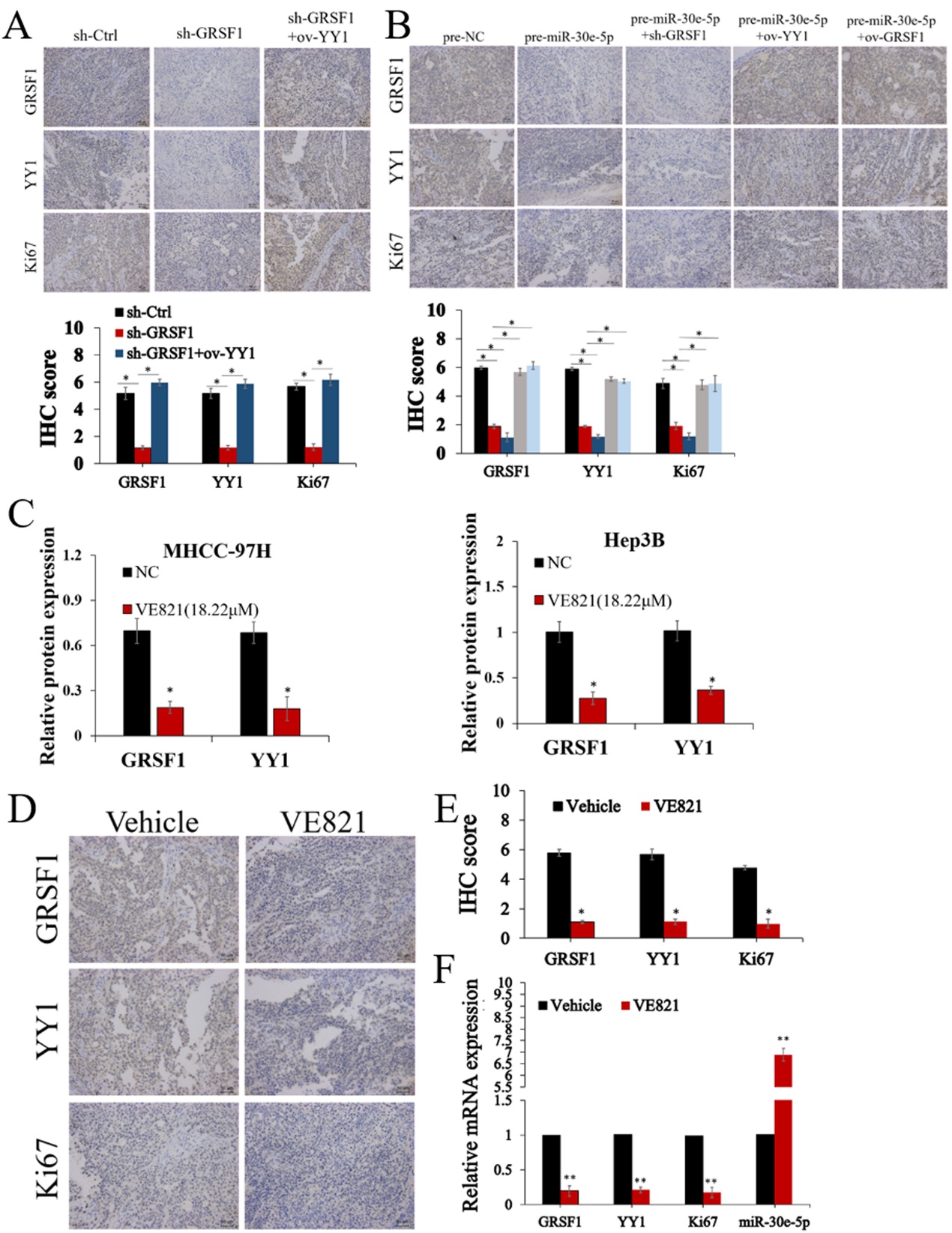


**Fig. S4. A. B.** IHC assay showing GRSF1, YY1 and Ki67 expression in xenograft tumor tissue (n=5, magnification, 200×). **C.** Western blot analysis of GRSF1 and YY1 expression in HCC cells. **D.** IHC assay showing GRSF1, YY1 and Ki67 expression in xenograft tumor tissue in the VE821 and vehicle groups. **E.F.** GRSF1, YY1, Ki67 and miR-30e-5p expression in xenograft tumor tissue from the VE821 and vehicle groups. (magnification, 200×). Values are the mean± SEM (n=5). **p*<0.05, ***p*<0.01.

**Fig. S5**


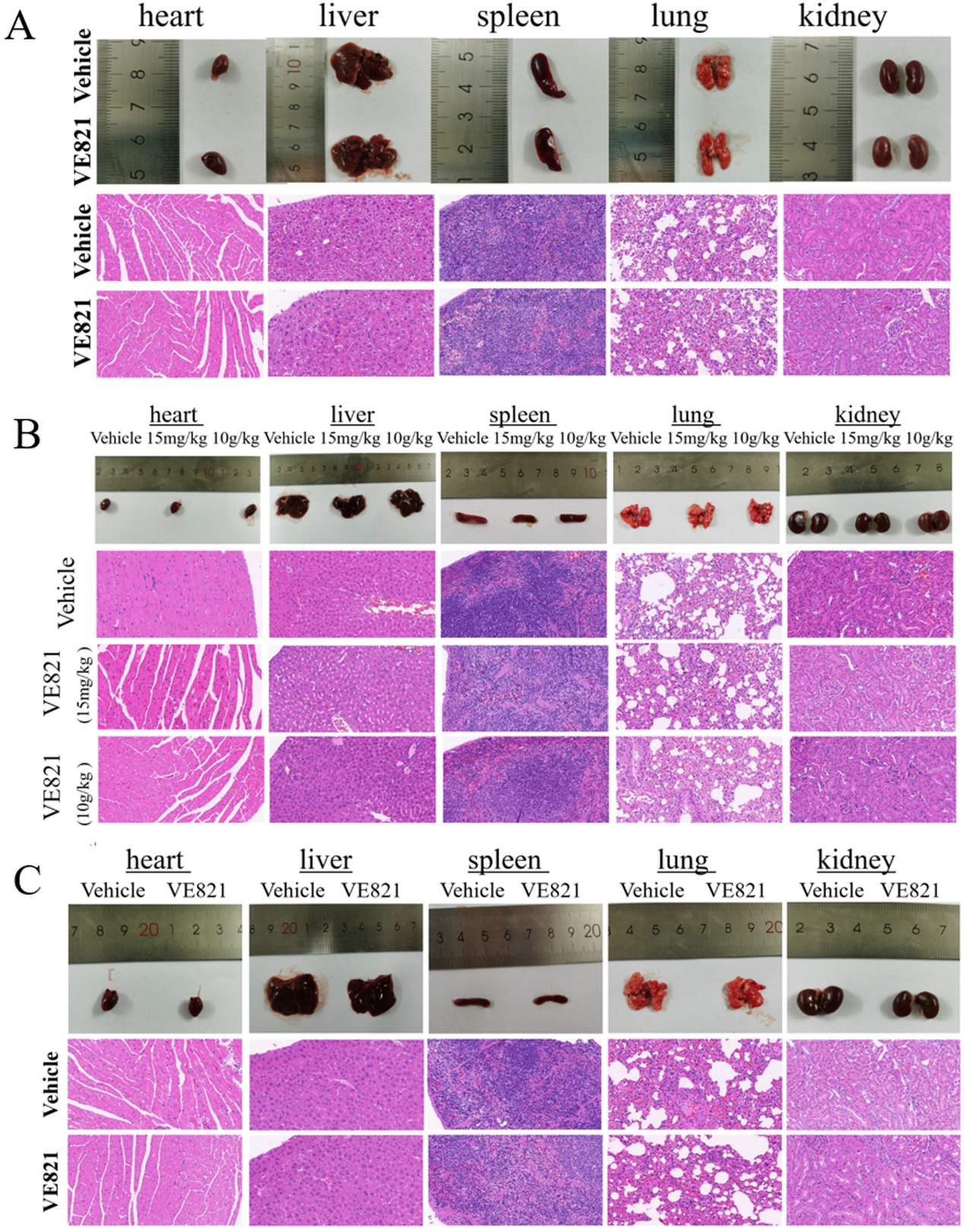


**Fig. S5. A.** HE staining of the major organs in tumor-bearing immunodeficient mice (magnification, 200×). **B. C.** HE staining of the major organs in healthy mice (n=5, magnification, 200×). Values are the mean± SEM (n=5). **p*<0.05, ***p*<0.01.

**Fig. S6**


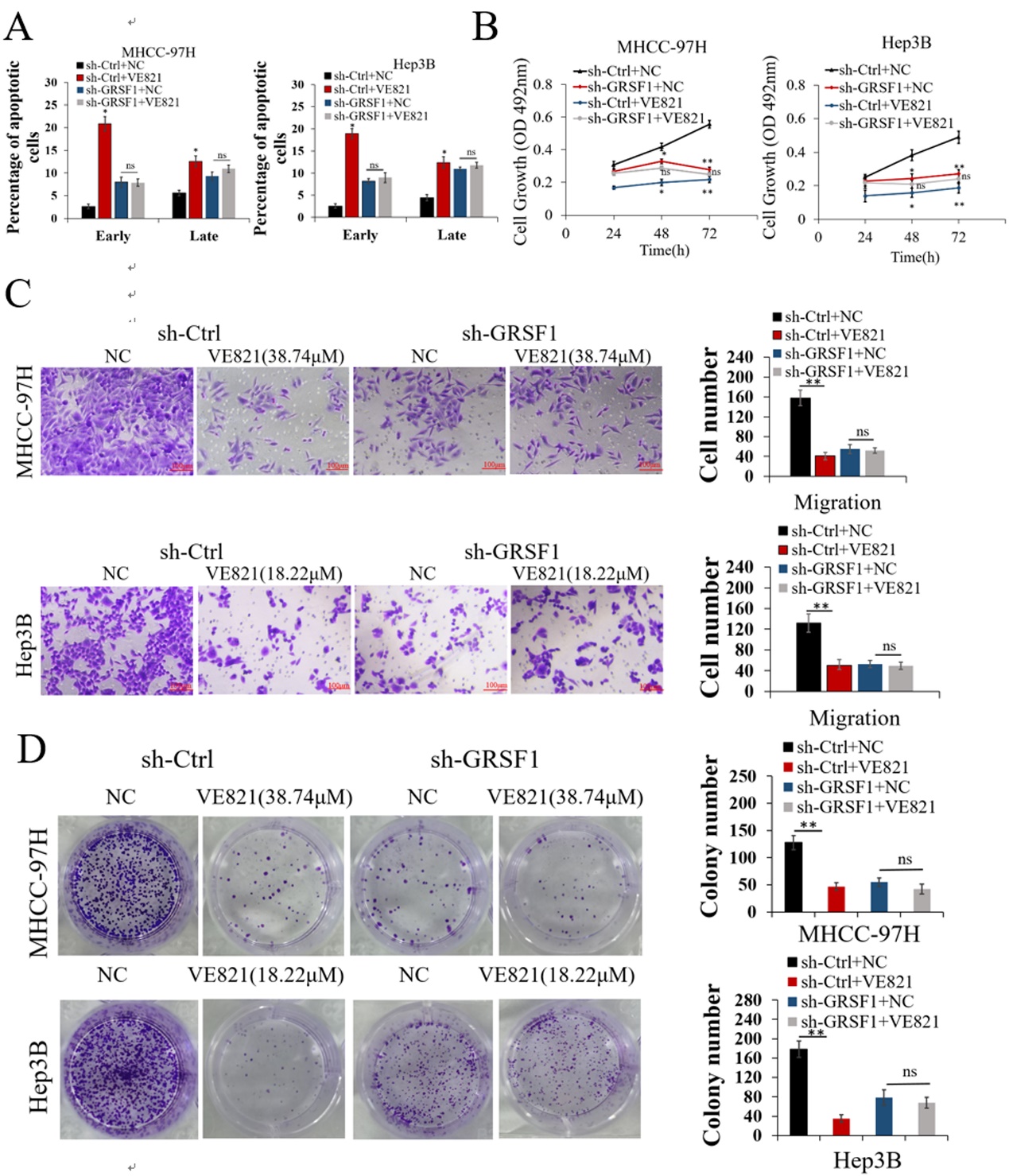


**Fig. S6.A.** VE821 enhanced the apoptosis of HCC cells transfected with sh-control vectors but did not further promote HCC cell apoptosis in the background of GRSF1 depletion. **B-D.** VE821 inhibited the malignant biological behavior of HCC cells transfected with sh-control vectors but did not further reduce GRSF1-deficient HCC cell proliferation (**B**) migration (**C**) or colony formation (**D**). The invaded cells in Transwell assays were quantified by counting the cells in 10 random fields (magnification, 200×). Values are the mean± SEM (n=3) **p*<0.05, ***p*<0.01.

**Fig. S7**


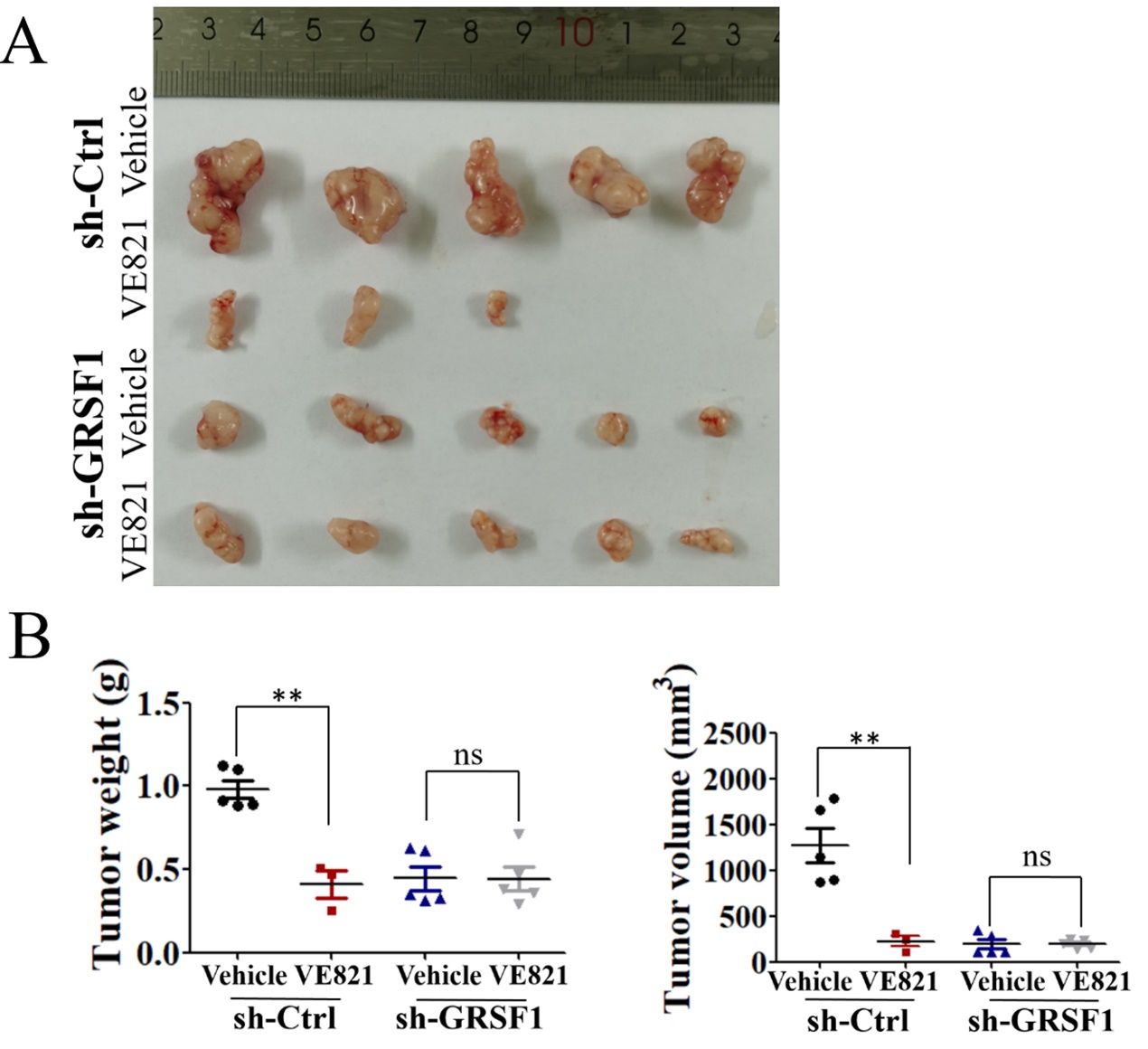


**Fig. S7.** VE821 suppressed HCC xenograft tumor growth but did not further inhibit tumor growth in the background of GRSF1 depletion (n=5). ***p*<0.01
